# Investigating confounding in network‐based test‐negative design influenza vaccine effectiveness studies—Experience from the DRIVE project

**DOI:** 10.1111/irv.13087

**Published:** 2022-12-22

**Authors:** Anke L. Stuurman, Miriam Levi, Philippe Beutels, Hélène Bricout, Alexandre Descamps, Gaël Dos Santos, Ian McGovern, Ainara Mira‐Iglesias, Jos Nauta, Laurence Torcel‐Pagnon, Jorne Biccler, DRIVE consortium

**Affiliations:** ^1^ P95 Epidemiology and Pharmacovigilance Leuven Belgium; ^2^ Centre for Health Economics Research and Modelling Infectious Diseases, Vaccine and Infectious Disease Institute University of Antwerp Antwerp Belgium; ^3^ Epidemiology Unit, Department of Prevention Tuscany Centre Health Authority Florence Italy; ^4^ Medical Department Sanofi Lyon France; ^5^ Inserm CIC 1417, Assistance Publique Hôpitaux de Paris, Hôpital Cochin Université de Paris Paris France; ^6^ Epidemiology, Value Evidence and Outcomes GSK Wavre Belgium; ^7^ Center or Outcomes Research and Epidemiology, Medical Affairs Seqirus Inc. Cambridge Massachusetts USA; ^8^ Vaccine Research Department Foundation for the Promotion of Health and Biomedical Research of Valencia Region (FISABIO – Public Health) Valencia Spain; ^9^ Department of Innovation & Development, Established Pharmaceuticals Division Abbott Healthcare Products B.V. Weesp The Netherlands

**Keywords:** adjustment, confounders, covariate, influenza vaccine effectiveness, test‐negative design

## Abstract

**Background**: Establishing a large study network to conduct influenza vaccine effectiveness (IVE) studies while collecting appropriate variables to account for potential bias is important; the most relevant variables should be prioritized. We explored the impact of potential confounders on IVE in the DRIVE multi‐country network of sites conducting test‐negative design (TND) studies.

**Methods**: We constructed a directed acyclic graph (DAG) to map the relationship between influenza vaccination, medically attended influenza infection, confounders, and other variables. Additionally, we used the Development of Robust and Innovative Vaccines Effectiveness (DRIVE) data from the 2018/2019 and 2019/2020 seasons to explore the effect of covariate adjustment on IVE estimates. The reference model was adjusted for age, sex, calendar time, and season. The covariates studied were presence of at least one, two, or three chronic diseases; presence of six specific chronic diseases; and prior healthcare use. Analyses were conducted by site and subsequently pooled.

**Results**: The following variables were included in the DAG: age, sex, time within influenza season and year, health status and comorbidities, study site, health‐care‐seeking behavior, contact patterns and social precautionary behavior, socioeconomic status, and pre‐existing immunity. Across all age groups and settings, only adjustment for lung disease in older adults in the primary care setting resulted in a relative change of the IVE point estimate >10%.

**Conclusion**: Our study supports a parsimonious approach to confounder adjustment in TND studies, limited to adjusting for age, sex, and calendar time. Practical implications are that necessitating fewer variables lowers the threshold for enrollment of sites in IVE studies and simplifies the pooling of data from different IVE studies or study networks.

## 1 INTRODUCTION

Continuous monitoring of influenza vaccines performance in real‐life settings is important to complement clinical trial data. Annual estimates of influenza vaccine effectiveness (IVE) are necessary, as influenza vaccines composition is reassessed every year and circulating influenza viruses differ from season to season. However, IVE is not only affected by circulating strains but also by the individual's immune response (modulated by age or other individual characteristics and history) and vaccine production technology and formulation. The DRIVE (Development of Robust and Innovative Vaccines Effectiveness), launched in 2017, is a public–private partnership set up to address the request of the European Medicine Agency (EMA) to provide annual effectiveness evaluation of all individual influenza vaccine brands commercialized in Europe.^1^ A large sample size is required to account for the diversity of influenza vaccines and vaccine recommendations. Therefore, establishing a large study network to conduct observational studies for IVE while collecting appropriate variables to account for potential bias is important.

Observational studies of seasonal IVE are susceptible to bias and confounding, and these factors need to be considered at the study design and analysis stages. Differences in disease risk or in care‐seeking behaviors between vaccinated and unvaccinated subjects and the difference in the probability of being vaccinated can substantially bias IVE estimates. Even when reducing selection bias, the true IVE may be overestimated or underestimated whenever confounding is present. Several strategies are available to prevent or at least reduce bias and confounding by known factors, such as restriction of the study population (e.g., to persons seeking care for respiratory symptoms in test‐negative design (TND) case‐control studies or to persons for whom a nasal swab was collected within a predefined number of days of symptom onset), stratification of estimates (e.g., by age group or population subgroups), matching, and statistical adjustment through multivariate regression.^2^

Nevertheless, in order to make a network successful and sustainable on a large scale, tradeoffs should be made in the collection of the most relevant variables. Throughout the 5 years of the DRIVE project, field‐based experts from both public and private sectors have discussed which confounders should be integrated into the generic protocol and collected by sites to ensure robust IVE estimates. EMA scientific advice was also sought by the DRIVE partners on the required number of confounders and the relevance of a parsimonious analysis. For IVE analyses in the 2017/2018 influenza season, the DRIVE took advantage of data collected through existing infrastructures and selected confounders through model building.^3^ In 2018/2019, the first season for which a common protocol was developed and used, IVE estimates were adjusted for a fixed, elaborate set of confounders, namely age, sex, calendar time, presence of at least one chronic condition, pregnancy, number of hospitalizations or General Practitioner (GP) visits in the previous year, and vaccination status in previous season.^4^ However, some sites were not able to collect all variables, either not at all or not for all subjects. This led to inconsistent confounder adjustment across sites and exclusion of subjects with missing values. To harmonize confounder adjustment and minimize data loss, the number of covariates adjusted for was decreased as of the 2019/2020 season, retaining only age, sex, and calendar time.^5^ Parsimonious confounder adjustment was supported by a post hoc analysis of the DRIVE's 2018/2019 data and was previously proposed by Lane et al according to an analysis of data from the Victorian Influenza Sentinel Practice Network in Australia^6^ and is in line with findings from the Canadian Sentinel Practitioner Surveillance Network (CSPSN).^7^

However, we aimed to further explore the impact of potential confounders on IVE in the context of a multi‐country TND network and to check through an in‐depth multi‐season secondary analysis if our previously chosen parsimonious confounder adjustment strategy could be justified. We constructed a DAG to map the most relevant IVE confounders and other variables. We used the DRIVE data from the 2018/2019 and 2019/2020 seasons to understand the role of covariates as predictors of vaccination status and case status, and to explore the effect of covariate adjustment on IVE point estimates.

## 2 METHODS

### 2.1 DAG

We constructed a directed acyclic graph (DAG) to visually represent the relationship between influenza vaccination and medically attended laboratory‐confirmed influenza infection presenting as influenza‐like illness (ILI) or severe acute respiratory infection (SARI). DAGs are visual tools used to identify confounding variables and common (including unmeasured/unmeasurable) causes of the exposure and outcome and to explicitly state the assumptions made regarding relationships between variables.^8^ The full causal diagram, describing all underlying relationships among all possible variables, is often not known. However, in 2011, VanderWeele et al used the so‐called “disjunctive cause criterion” to demonstrate that controlling for all observed variables that affect the exposure, the outcome or both are sufficient to control for confounding.^9^

We built upon the DAG that Lane et al developed by taking covariates used in >10% of published TND studies identified in a systematic review.^6^ Potential sources of confounding were identified from a systematic review on bias and confounding,^10^ a systematic review on determinants of influenza vaccination uptake in older adults,^11^ and expert input, and were included regardless of the operational feasibility of data collection. Using DAGitty, a browser‐based environment for creating, editing, and analyzing causal diagrams,^12^ we tested the minimal sufficient adjustment set for estimating the total effect of current influenza vaccination on medically attended influenza infection (i.e., for DAG closure).

### 2.2 Dataset

This exploratory analysis made secondary use of the DRIVE datasets, based on TND studies conducted at four GP sites and four hospital sites in five countries in the 2018/2019 influenza season and at four GPs sites and eight hospital sites in seven countries in the 2019/2020 influenza season (Table 1). Subject characteristics are presented in supporting information 1. While the DRIVE covered the influenza seasons from 2017 to 2022, data from the other DRIVE seasons were not used because common protocols had not yet been implemented in 2017/2018, the number of influenza cases in Europe was historically low as a result of the COVID‐19 pandemic in 2020/2021 and, data collection for the 2021/2022 season was still ongoing at the time of the analysis.

**TABLE 1:** Overview of sites and number of subjects (cases and controls) per site and setting in the 2018/2019 and 2019/2020 influenza seasons DRIVE data

		Number of subjects (%)					
		Season 2018/2019			Season 2019/2020		
Country	Site name	Children 6 m to 17y	Adults 18–64y	Older adults ≥65y	Children 6 m to 17y	Adults 18–64y	Older adults ≥65y
**Primary care (ILI** ^**a**^ **)**							
Austria	Medical U Vienna	432 (21)	422 (21)	33 (8)	639 (27)	673 (30)	47 (14)
Italy	CIRI‐IT GP network	384 (19)	520 (26)	190 (45)	698 (29)	524 (23)	146 (42)
Italy	ISS	1149 (57)	1022 (50)	178 (42)	938 (40)	863 (38)	119 (34)
England	RCGP	45 (2)	72 (4)	20 (5)	97 (4)	185 (8)	35 (10)
*Total*		*2010*	*2036*	*421*	*2372*	*2245*	*347*
**Hospital (SARI** ^**b**^ **)**							
Finland	Helsinki UH, Jorvi H	‐	103 (9)	171 (8)	‐	56 (5)	69 (4)
France	I‐REIVAC	‐	‐	‐	‐	134 (13)	246 (15)
Italy	CIRI‐IT BIVE	820 (51)	278 (25)	500 (23)	770 (55)	296 (28)	584 (35)
Romania	IBI Matei Bals	518 (32)	356 (33)	153 (7)	500 (36)	221 (21)	78 (5)
Spain	FISABIO	187 (12)	234 (21)	1099 (50)	19 (1)	157 (15)	486 (29)
Spain	Germans Trias i Pujol UH	‐	‐	‐	25 (2)	68 (6)	89 (5)
Spain	La Paz UH	‐	‐	‐	‐	15 (1)	21 (1)
Spain	Vall d'Hebron UH	70 (4)	124 (11)	271 (12)	78 (6)	110 (10)	100 (6)
*Total*		*1595*	*1095*	*2194*	*1392*	*1057*	*1673*

*Note*: BIVE: Italian Hospital Network; CIRI‐IT: Interuniversity Research Center on Influenza and other Transmissible Infections; GP: General Practitioner; FISABIO: Foundation for the Promotion of Health and Biomedical Research of the Valencia Region; H: hospital; I‐REIVAC: Innovative Clinical Research Network In Vaccinology; IBI Matei Bals: National Institute for Infectious Diseases “Prof. Dr. Matei Balş”; ILI: influenza‐like illness; ISS: Italian National Institute of Health; RCGP: Royal College of General Practitioners Research and Surveillance Centre; SARI: severe acute respiratory infection; U: University; UH: University Hospital.

^a^ ILI was defined as an individual presenting with sudden onset of symptoms; AND at least one of the following systemic signs or symptoms: fever/feverishness, malaise, headache, and myalgia; AND at least one of the following respiratory symptoms: cough, sore throat, and shortness of breath.

^b^ SARI was defined as a hospitalized person with a suspicion of infection with at least one of the systemic signs or symptoms defined above or deterioration of general condition; AND at least one of the respiratory symptoms defined above, at admission or within 48 h after admission.

Data collection and site characteristics have been previously described more in depth elsewhere.^4^, ^5^ In brief, patients with ILI were enrolled in the primary care setting and patients with SARI in the hospital setting (see Table 1 for definitions). Only community‐dwelling ILI and SARI patients presenting during the study period for analysis, ≤7 days after symptom onset and without contraindication for influenza vaccination, were included in the dataset. The outcome of interest was laboratory‐confirmed influenza (primarily through reverse transcription polymerase chain reaction [RT‐PCR]). The exposure of interest was any seasonal influenza vaccine (>14 days prior to symptom onset) in the respective season.

### 2.3 Covariates considered

Covariates selected for this study had to be present in the DAG, be collected by at least 50% of the DRIVE sites in 2019/2020, and have a prevalence of at least 10% in one of the age groups. This was done as variables with a low prevalence are unlikely to lead to a large change in the coefficients. The following covariates fulfilled these criteria and were included: age; sex; calendar time (i.e., week of symptom onset of current ILI/SARI episode); absence or presence of at least one, two, or three chronic diseases (up to 12 chronic disease types were considered; supporting information 2); presence of specific chronic diseases (cancer, cardiovascular disease, diabetes, lung disease, renal disease, and obesity); number of hospitalizations in the last year (0, 1–2, >2); and number of primary care visits in the last year (0, 1–5, >5). Smooth functions of age and calendar time were modeled by penalized (10‐dimensional) cubic regression splines^13^ estimated using restricted maximum likelihood for smoothness selection.^14^ In case fewer than 10 unique age or onset date values were observed, the effect of age or onset date was modeled using a linear function instead.

Data on age, sex, date of symptom onset, and the presence of at least one chronic condition were available for all sites/seasons, whereas availability of specific chronic diseases and prior healthcare use varied across sites (supporting information 3).

### 2.4 Predictors of vaccination and the outcome

Seasonal influenza vaccines are preferentially assigned to specific population groups according to their specific indication (different vaccines are recommended depending on age and/or on the presence of medical conditions) and according to national or regional vaccine recommendations,^15^, ^16^ reflecting known risk factors for medically attended influenza in the general population. However, it is less clear whether these covariates are predictors for laboratory‐confirmed influenza (“being a case”) among ILI/SARI patients included in TND studies, where the comparator group consists of medically attended non‐influenza ILI/SARI patients. We used logistic regression to identify which covariates were associated with vaccination among test‐negative controls (as a proxy for the general population) and which covariates were associated with influenza among ILI/SARI patients, by vaccination status. Odds ratios (OR) adjusted for age, sex, calendar time, site, and season were calculated. Analyses were stratified by age (6 months (m) to 17 years (y), 18 to 64 y, ≥65 y) and setting (hospital vs primary care).

### 2.5 Quantifying potential confounding effects

Following the convention used in TND studies, IVE was defined as 100 * (1 – OR), where the OR is the ratio of the odds of being a test‐positive case among the vaccinated compared to the odds of being a test‐positive case among the unvaccinated. The effect of each covariate on the IVE was explored. Reference models adjusted for age, sex, calendar time, and season were built. Age, sex, and calendar time are frequently adjusted for covariates in IVE studies.^2^, ^17^ The covariate “influenza season” was included, as the analysis used data from two influenza seasons (2018/2019 and 2019/2020).

Comparator models were adjusted for one additional covariate (see section covariates considered). The absolute and relative changes in IVE obtained through comparator models versus the reference models (and for the reference model compared to a model adjusted for season only) were described. Absolute changes were calculated as IVE _comparator model_ − IVE _reference model_. Relative changes were calculated as IVE _comparator model_/IVE _reference model._ Relative changes of >10% were considered meaningful.^6^, ^18^, ^19^, ^20^ Conditional logistic regression models were fitted (similar to Lane et al^6^) on the site and season level for each age group, and the changes in IVE estimates were pooled by setting through random‐effects meta‐analysis using the Hartung–Knapp–Sidik–Jonkman method.^21^ Confidence intervals for the change‐in‐estimate parameters were constructed using non‐parametric bootstrap.^22^ Only the pooled (and not the site‐specific) results are presented.

To understand the minimum strength of association (on the risk ratio scale) that an unmeasured confounder would need to have with the vaccination and/or outcome, conditional on the covariates in the reference model, to fully explain away the observed IVE, E‐values were calculated (post‐hoc).^23^, ^24^ E‐values were calculated for the pooled IVE point estimates, by age and setting, and corresponding CI limited closest to 0.

## 3 RESULTS

**FIGURE 1:**
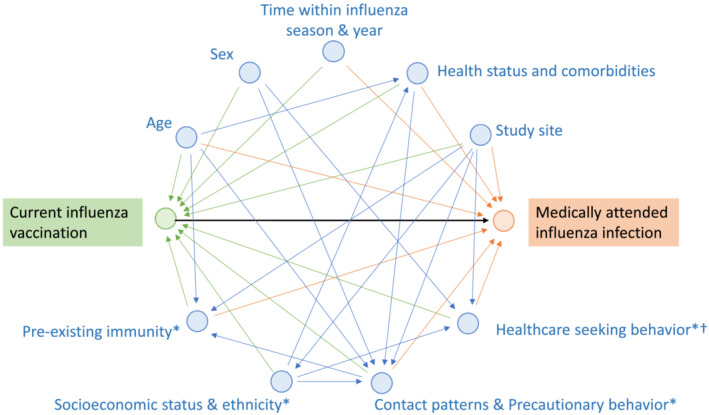
Directed acyclic graph showing the relationship between current influenza vaccination and medically attended influenza infection. Exposure and arrows pointing toward the exposure are shown in green, outcome and arrows pointing toward the outcome in orange, other variables and arrows in blue. Adapted from Lane *et al*.^6^ * indicates the variable was not included in the DAG by Lane *et al*. † Healthcare seeking behavior: In addition to whether or not an individual seeks care, the timing of care seeking is important and applying time‐related restriction factors (regarding too long interval since symptom onset, too short interval since vaccination, and presentation when influenza is not circulating) is recommended. DAG, directed acyclic graph

A DAG was constructed (Figure 1). We were in agreement with the rationale for including the covariates age, sex, calendar time within the influenza season (month, week etc.), health status and comorbidities, study site, and year in the DAG described by Lane *et al* ^6^ Additionally, Lane *et al* describe the exclusion (restriction) criteria applied to the study population (too long interval between onset and specimen collection, presenting outside influenza risk period, and symptom onset <15 days after vaccination).^6^ We were also in agreement with these criteria; and they were part of the DRIVE study's inclusion/exclusion criteria. In our DAG, we combined health status, non‐immunocompromising comorbidities, and immunocompromising comorbidities into one confounder, as we considered comorbidities to be part of “health status,” and we considered both types of comorbidities to impact the outcome (whereas in the DAG by Lane *et al*, only immunocompromising conditions are linked to the outcome). Examples of ways that health status and comorbidities could be operationalized include looking at the presence of comorbidities that lead to a recommendation for influenza vaccination, the presence of individual comorbidities, number of comorbidities, frailty, and number of recent hospitalizations. Four additional covariates were included in our DAG: healthcare‐seeking behavior, pre‐existing immunity from infection or vaccination, social contact patterns and precautionary social behavior, and socioeconomic status. The rationale for their inclusion is described in depth in Table 2. In order to be classified as a case in a TND study, an individual has to be exposed to the influenza virus, develop ILI or SARI, seek medical care, be tested for influenza, and have a positive test result. The confounders associated with the outcome in the DAG may impact one or more of these steps.

**TABLE 2:** Rationale for inclusion of confounders in the DAG

Confounder	Rationale for inclusion in the DAG
Healthcare‐seeking behavior	In TND studies, there is less bias because of healthcare‐seeking behavior, defined as a person's propensity to seek care when ill, than in cohort studies, as the study population is restricted to persons who seek medical care for ILI or SARI [45]. However, as described by Sullivan et al., “someone's propensity to seek care, […] is based on many factors” and is therefore “unlikely to be completely captured by a single binary indicator of whether or not a person presents himself/herself to a physician when experiencing influenza symptoms, so healthcare‐seeking would remain partially unobserved and the TND design is unlikely to completely block the effects of this confounder” [46]. Healthcare‐seeking behavior, in general, may also be associated with increased opportunities to be offered influenza vaccine. Healthcare‐seeking behavior bias is likely more pronounced for mild disease than for severe disease. Healthcare‐seeking behavior is not straightforward to operationalize, but proxies could include sex (with females being generally more prone to seek care than males^25^), the number of recent GP visits, or up‐to‐date pneumococcal vaccination (for adult age groups).^19^
Pre‐existing immunity from infection or vaccination	Depending on the circulating influenza strains and the degree and duration of residual immunity, persons having recently experienced an influenza infection may be (partially) protected from influenza [47, 48]. At the same time, a recent prior influenza infection has been reported by GPs as a factor that increases influenza vaccine acceptance [49]. Confounding because of immunizing infections may be expected to vary across seasons, as population‐level intensity, severity of recent influenza seasons and changes in influenza vaccine composition could impact the perceived necessity of vaccination. Prior influenza vaccination may be a confounder of IVE when influenza vaccination in the current season is associated with vaccination history and when vaccination modifies the risk of natural infection because of lower previous risk of infection or persisting immunity [50]. However, prior influenza vaccination is highly predictive of influenza vaccination in the current season^29^; this collinearity may lead to overadjustment if this variable is included in statistical models.
Social contact patterns and precautionary social behavior	Social contact patterns affect the risk of exposure to influenza virus. Social contact patterns may be related to occupation; healthcare workers with direct patient contact may be more likely to have occupational exposure to influenza, and this group is typically targeted for influenza vaccination [51]. Contact patterns have been highly associated with age and household size, whereas the average number of contacts varies between countries [52–54]. Persons working with young children may be more willing to accept vaccination if they have an additional risk factor (e.g., a medical condition). Among older adults, social inclusion into family or informal social networks—which may increase their number of contacts—was found to positively affect vaccine uptake.^11^ In a study conducted among older adults in three European countries, exposure to children under the age of five living outside of the household explained 10% of all acute respiratory tract infections [55]. Precautionary social behavior affects the risk of exposure to influenza virus and may impact motivation to be vaccinated. Although precautionary behavior is always relevant in the prevention of influenza, preventive measures such as face mask wearing, physical distancing, and handwashing have become widespread since 2020 with the COVID‐19 pandemic. These measures against SARS‐COV‐2 virus transmission also impact the circulation of other respiratory viruses such as influenza, as illustrated by the strong reductions in influenza circulation in Europe in the 2020/2021 Northern Hemisphere winter [56]. In addition, precautionary behavior such as mask wearing and distancing likely lead to a smaller dose of the initial inoculum if exposed despite the measures taken, thereby reducing the chance of developing severe disease [57]. The relevance of precautionary social behavior in IVE studies will likely depend on future COVID‐19 containment measures.
Socioeconomic status and ethnicity	Higher socioeconomic status or educational level may support increased vaccine uptake (in older adults),^11^ and uptake has been found to be lower in certain ethnic groups (migration background, religion) [58–61]. At the same time, it may impact healthcare‐seeking behavior (including accessibility of healthcare) and other social aspects such as contact patterns and health beliefs leading to precautionary behavior, and health status.

*Note*: GP: General Practitioner; DAG, directed acyclic graph; TND, test‐negative design; ILI, influenza‐like illness; SARI, severe acute respiratory infection; IVE, influenza vaccine effectiveness.

The minimal sufficient adjustment sets for estimating the total effect of current influenza vaccination on medically attended influenza infection include all confounders except for sex and socioeconomic status, as their relationship with the outcome is mediated via other confounders. Note that despite this finding, we decided to include sex in our reference model for further analyses as data on multiple other confounders (for which sex may serve as a partial proxy, such as healthcare‐seeking behavior or contact with young children^25^, ^26^) are absent.

### 3.1 Predictors of vaccination status and the outcome

**FIGURE 2:**
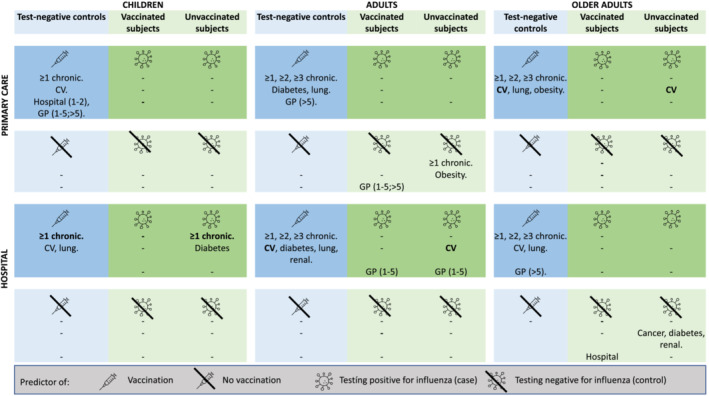
Predictors of vaccination among test‐negative controls and predictors of testing positive for influenza (case) and testing negative for influenza (control), by vaccination status, based on odds ratios adjusted for age, sex, calendar time, and season that do not include one in the 95%CI. Variables that are predictors of both vaccination status and the outcome are marked in bold. The covariates included in the analyses were presence of at least one, at least two, and at least three chronic diseases (≥1, ≥2, ≥3 chronic); presence of cancer, cardiovascular disease, diabetes, lung disease, renal disease, and obesity; and number of hospitalizations and primary care visits in the last year.’‐’ signifies no significant predictors.

Among the 11 tested covariates (presence of chronic conditions [≥1, ≥2, ≥3], cancer, cardiovascular disease, diabetes, lung disease, obesity, renal disease, number of hospitalizations, and number of GP visits in the past year) all except cancer were predictive (adjusted odds ratio [aOR] significantly different from 1) of influenza vaccination among test‐negative controls in at least one age group/setting combination (Figure 2). The number and percentage of subjects with each covariate by vaccination status and the aORs are presented in supporting information 4. The presence of at least one chronic condition was predictive of vaccination across all age groups and settings, with aORs ranging from 2.3 (95%CI 1.5–3.7) among older adults in primary care to 4.5 (95%CI 2.9–7.0) among children in the hospital setting. No trends across age groups regarding aOR of the number of chronic conditions were observed. Cardiovascular disease was the strongest predictor in children (aOR 10.5 [95%CI 1.6–70.4] in the primary care and 6.3 [95%CI 2.8–14.3] in the hospital settings). In adults, the strongest predictors were the number of GP visits and lung disease in the primary care setting (aOR 3.9 [95%CI 2.0–7.3] and 3.9 [2.5–6.2], respectively) and the presence of at least one chronic condition in the hospital setting (aOR 2.5 [95%CI 1.8–3.7]). Obesity (aOR 4.7 [95%CI 1.4–15.5]) and at least five GP visits were the strongest predictors in older adults in the primary care and hospital settings, respectively. No predictors of non‐vaccination were identified.

Predictors of ILI/SARI subjects testing positive versus negative for influenza were evaluated by vaccination status (Figure 2). The number and percentage of cases and controls with each covariate and the ORs are presented in supporting information 5. The majority of statistically significant predictors were identified among unvaccinated subjects. The presence of at least one chronic condition in children in the hospital setting and cardiovascular diseases among adults in the hospital setting and older adults in the primary care setting were predictors of both the vaccination status and outcome in the unvaccinated.

### 3.2 Relative change in IVE estimate

Absolute and relative changes in IVE of comparator models versus the reference models are shown in Table 3 (in the primary care setting) and Table 4 (in the hospital setting). One comparator model resulted in *a* > 10% relative change of the IVE point estimate: for older adults in the primary care setting, additionally adjusting for lung disease increased the IVE estimate by 15%.

**TABLE 3:** Absolute and relative changes in IVE estimate when adjusting for an additional covariate compared to the reference model adjusted for age, sex, calendar, and season, by age group in the primary care setting

	Change in IVE estimate					
	Children		Adults		Older adults	
	Absolute % Δ	Relative Δ (ref = 1.0)	Absolute % Δ	Relative Δ (ref = 1.0)	Absolute % Δ	Relative Δ (ref = 1.0)
**Chronic conditions**						
≥ 1	0.1 (−0.6; 0.7)	1.00 (0.98; 1.02)	2.6 (−8.1; 13.3)	1.02 (0.91; 1.15)	1.4 (−44.0; 46.9)	1.02 (0.90; 1.16)
≥ 2	0.0 (−0.0; 0.0)	NA (NA; NA)	0.6 (−2.2; 3.5)	1.01 (0.97; 1.05)	0.7 (−7.5; 8.8)	1.01 (0.88; 1.15)
≥ 3	NA (NA; NA)	NA (NA; NA)	0.4 (−0.5; 1.3)	1.01 (0.99; 1.02)	−1.4 (−14.0; 9.8)	0.97 (0.67; 1.41)
**Specific conditions**						
Cancer	−0.2 (−0.5; 0.3)	0.99 (0.98; 1.01)	0.2 (−1.6; 1.9)	1.00 (1.00; 1.01)	0.0 (−8.9; 8.8)	1.00 (0.93; 1.08)
Cardiovascular disease	−0.7 (−2.0; 0.4)	0.97 (0.92; 1.01)	2.0 (−8.8; 12.8)	1.04 (1.00; 1.07)	−9.2 (−25.0; 8.2)	0.90 (0.77; 1.06)
Diabetes	‐	‐	5.2 (−21.7; 32.1)	1.03 (0.79; 1.34)	1.7 (−9.9; 11.7)	1.02 (0.93; 1.11)
Lung disease	−0.0 (−1.3; 1.2)	1.00 (0.97; 1.03)	−1.8 (−23.8; 20.3)	1.01 (0.71; 1.44)	14.0 (−8.6; 34.8)	**1.15 (1.00; 1.34)**
Obesity	−0.0 (−0.2; 0.2)	1.00 (0.99; 1.01)	−0.4 (−12.8; 12.0)	0.99 (0.82; 1.21)	2.2 (−21.1; 20.7)	1.02 (1.01; 1.04)
Renal disease	‐	‐	0.3 (−6.2; 6.8)	1.00 (0.90; 1.12)	−0.6 (−6.8; 4.0)	0.99 (0.94; 1.04)
**Healthcare use in past year**						
Hospitalizations	0.3 (−0.1; 0.7)	1.01 (0.99; 1.03)	−0.7 (−10.9; 9.5)	0.99 (0.87; 1.13)	1.0 (−0.7; 2.8)	1.01 (0.98; 1.04)
GP visits	−1.3 (−4.2; 1.4)	0.98 (0.93; 1.03)	2.4 (−2.2; 6.6)	1.04 (0.96; 1.12)	0.2 (−12.2; 11.3)	1.00 (0.87; 1.15)

*Note*: GP: General Practitioner; IVE, influenza vaccine effectiveness.

**TABLE 4:** Absolute and relative changes in IVE estimate when adjusting for an additional covariate compared to the reference model adjusted for age, sex, calendar, and season, by age group in the hospital setting

	Change in IVE estimate					
	Children		Adults		Older adults	
	Absolute Δ (%)	Relative Δ	Absolute Δ (%)	Relative Δ	Absolute Δ (%)	Relative Δ
**Chronic conditions**						
≥ 1	−11.8 (−57.4; 33.8)	0.92 (0.54; 1.59)	−0.5 (−5.0; 4.0)	0.99 (0.93; 1.07)	−0.5 (−1.8; 0.8)	0.99 (0.98; 1.01)
≥ 2	0.1 (−10.8; 11.0)	1.00 (0.88; 1.15)	−1.4 (−4.5; 1.6)	0.98 (0.93; 1.03)	0.2 (−1.1; 1.4)	1.00 (0.98; 1.03)
≥ 3	−0.1 (−0.5; 0.3)	1.00 (0.99; 1.01)	0.5 (−2.7; 3.8)	1.01 (0.97; 1.05)	0.3 (−0.3; 1.0)	1.01 (0.99; 1.02)
**Chronic condition category**						
Cancer	−0.5 (−0.9; −0.1)	0.99 (0.98; 1.00)	−0.7 (−2.3; 0.9)	0.99 (0.97; 1.01)	−0.2 (−0.9; 0.5)	1.00 (0.99; 1.01)
Cardiovascular disease	−0.7 (−24.3; 23.0)	1.00 (0.78; 1.28)	−1.1 (−3.2; 0.9)	0.99 (0.96; 1.02)	0.0 (−1.5; 1.5)	1.00 (0.98; 1.03)
Diabetes	0.1 (−7.2; 7.3)	1.00 (0.91; 1.10)	−0.2 (−3.3; 2.9)	1.00 (0.96; 1.04)	0.2 (−1.1; 1.4)	1.00 (0.98; 1.03)
Lung disease	−1.1 (−57.8; 55.6)	0.98 (0.49; 1.98)	0.6 (−2.5; 3.8)	1.01 (0.96; 1.05)	−0.1 (−1.0; 0.8)	1.00 (0.98; 1.01)
Obesity	−0.2 (−1.6; 1.2)	1.00 (0.98; 1.02)	−0.6 (−1.7; 0.5)	0.99 (0.98; 1.00)	−0.1 (−1.5; 1.3)	1.00 (0.98; 1.02)
Renal disease	−5.5 (−73.6; 62.7)	0.99 (0.65; 1.49)	−0.2 (−1.7; 1.4)	1.00 (0.96; 1.03)	−0.9 (−3.1; 1.2)	0.99 (0.97; 1.02)
**Healthcare use in past year**						
Hospitalizations	−2.3 (−5.1; 0.4)	0.96 (0.88; 1.04)	−1.3 (−3.8; 1.2)	0.99 (0.95; 1.03)	−1.1 (−5.0; 2.9)	1.00 (0.96; 1.04)
GP visits	−1.1 (−8.2; 5.1)	0.96 (0.77; 1.17)	−0.6 (−82.0; 80.8)	1.00 (0.79; 1.27)	1.6 (−4.8; 8.0)	1.03 (0.96; 1.10)

GP: General Practitioner; IVE, influenza vaccine effectiveness.

Results for the reference model versus the model adjusted for season only are shown in supporting information 6. In the primary care setting, adjusting for calendar time increased the IVE estimate by 16% among children and 24% among older adults.

A sensitivity analysis in which reference models were adjusted for age, sex, time, season, and presence of at least one chronic condition was carried out (supporting information 7). Similar to the main analysis, additionally adjusting for lung disease led to a 12% relative increase of the IVE estimate among older adults in the primary care setting.

As a secondary exploratory analysis, the analysis was repeated using a propensity‐score weighted model (see supporting information 8 for details on methods). Findings were very similar to the conditional model; the only covariate that led to *a* > 10% relative change in the IVE estimate was lung disease in older adults in the primary care setting, which led to a 16% increase.

E‐values for the IVE point estimate ranged from 1.3 (for older adults in the primary care setting) to 4.0 (for children in the primary care setting) (supporting information 9). This means that the observed IVE of 6.3% in older adults and 56.5% in children in the primary care setting could be explained away by an unmeasured confounder that was associated with influenza vaccination and/or the outcome by a risk ratio of at least 1.3‐fold and 4.0‐fold, respectively.

## 4 DISCUSSION

In our study, based on data collected in the 2018/2019 and 2019/2020 influenza seasons, the results showed that additionally adjusting for chronic conditions or prior healthcare use in site‐ and age‐specific models adjusted for age, sex, calendar time, and season did not lead to meaningful changes in the relative IVE estimate. This justifies a simple, harmonized approach to confounder adjustment in TND studies.

The only exception was the variable “lung disease,” adjusting for which increased the relative IVE by 15% among older adults in the primary care setting. Close to the threshold was also “cardiovascular disease” in the same population, decreasing the relative IVE by 10%. It would be of interest to know if these findings can be replicated in other VE networks. In previous research, Foppa *et al* found that not adjusting for chronic cardiopulmonary conditions could overestimate IVE if highly vaccinated controls with respiratory non‐ARI (acute respiratory infection) exacerbations were included in the study.^27^ This highlights the difference between risk factors for influenza in the general population (such as in cohort studies) and among patients seeking care for respiratory symptoms (such as in TND studies). For TND studies to identify risk factors for case status, these risk factors must be “either [be] totally distinct or clearly different in magnitude from the risk factors of illnesses that manifest with similar symptoms”.^28^

In both primary care and hospital settings, the presence of at least one chronic condition was a stronger predictor of vaccination among children than in the other age groups. This finding was expected, as vaccination was recommended and free in 2018/2019 and 2019/2020 for all children ≥6 months only for one site (in England) part of the DRIVE network; for other sites/countries, vaccination is recommended for children with comorbidities. By contrast, recommendations in adults also encompass occupational groups and all older adults are recommended for vaccination, thereby expecting to dilute chronic conditions as a predictor of vaccination.

The covariates available in the dataset used were insufficient for DAG closure; therefore, residual confounding cannot be ruled out. For some covariates on which we have no data, such as socioeconomic status, data collection may be feasible in IVE studies (even if practically challenging in some settings). Several of the other variables in the DAG are challenging to account for in IVE studies. For example, data collection on variables such as contact patterns and precautionary behavior, which require behavioral questionnaires ideally administered before symptom onset, is unlikely to be considered a priority in large IVE studies. Detailed data on prior potentially immunizing influenza episodes is not widely available as respiratory infections are frequently not medically attended or based on clinical diagnosis only. Although vaccination status in prior seasons is likely to be retrievable, it is highly collinear with influenza vaccination in the present season and its inclusion in the statistical model may therefore lead to overadjustment.^29^ The DAG describes the relationship between current influenza vaccination and medically attended laboratory‐confirmed influenza infection presenting as ILI/SARI, that is, test‐positive cases. Test‐negative controls, who present with non‐influenza respiratory infections, are not described in the DAG. However, IVE estimates can be biased by factors that modify non‐ILI ARI risk and many may be associated with influenza vaccination.^19^, ^27^, ^30^

Sullivan *et al* have reviewed the inclusion (and definition) of key variables in the principal analysis of 85 IVE studies and described how many studies applied the restriction criteria and adjusted for specific covariates.^17^ Clearly, there is important variation between studies. Examples of networks other than the DRIVE are the outpatient United States Flu VE Network, the United States Hospitalized Adults IVE Network (HAIVEN), the European I‐MOVE network, and the CSPSN, all of which adjusted IVE estimates for age and calendar time and accounted (through adjustment or restriction) for the interval from symptom onset to testing.^7^, ^31^, ^32^, ^33^, ^34^ The US Flu VE Network additionally adjusted for sex, self‐rated general health, race, and Hispanic ethnicity^31^; the HAIVEN for sex and race/ethnicity^34^; and I‐MOVE for sex, chronic conditions, and study site.^32^, ^33^ CSPSN found that additional adjustment for sex and presence of at least one comorbidity resulted in an absolute change of the IVE of ≤4% and, therefore, did not include these variables in their final model.^7^

The present study has several limitations. This was a secondary analysis of an existing dataset. Collection of data on chronic conditions (beyond “presence of at least one chronic condition”) and prior healthcare use were not mandatory and were consequently not available for all sites, sometimes only for a subset of subjects within one site. To optimize the use of all existing data, a complete case analysis was performed for each model. The drawbacks are, first, that the set of subjects included in each analysis was not identical, and second, that the number of conditions eligible to be counted to build the variables “presence of at least two or three chronic conditions” was not the same across all sites. Furthermore, the lack of more granular information on chronic conditions in the dataset, such as on the precise condition, disease severity, and frailty for older adults, precludes the possibility to assess in more detail the potential chronic conditions/diseases associated with the likelihood of being diagnosed with influenza.

There is no gold standard for the inclusion of chronic conditions in IVE studies. From a vaccination policy standpoint, the European Centre for Disease Prevention and Control (ECDC) provided a list of chronic conditions of importance to target the individuals with a higher risk of associated complications in case of influenza infection.^35^ Nevertheless, all conditions are considered equally likely to give rise to annual vaccination, although there may be a difference in proactive communication and vaccination by GPs, based on the specific condition of the patient. Furthermore, some studies demonstrated the importance of focusing on the frailty syndrome when assessing IVE, particularly in older adults.^36^ Frailty is a dynamic and multifactorial syndrome in older adults that represents a reduction in physiological reserve, limited ability to resist environmental stressors, and increased risk of functional decline. Frailty is a state of increased vulnerability to adverse outcomes compared to others of the same age.^37^ Applied studies highlighted the substantial confounding effect of frailty in the context of IVE studies and underscored the importance of using this multidimensional component instead of isolated factors to account for the health status and vulnerability of study participants.^38^

The statistical model used was a conditional model (also used in the DRIVE IVE studies^4^, ^5^), which models the effect of potential confounders on the outcome. A drawback of this model is that the non‐collapsibility of the odds ratio and incidence ratio implies that changes in the IVE because of the exclusion or inclusion of a covariate might not be caused by the confounding effect.^8^ This is more problematic for common outcomes (such as ILI) than for relatively rare outcomes (such as SARI).^39^, ^40^, ^41^ However, the findings based on the propensity‐score weighted model were very similar. Several assumptions underlie the statistical model, such as the absence of unmeasured confounders (which we consider unlikely, considering the DAG), that the effects take place on the logarithmic scale and that the effects are linear. We focused on the relative change rather than absolute change in IVE to have a more harmonized comparison across the age groups and settings. The 10% change threshold used to select meaningful covariates, though also commonly used by others,^6^, ^18^, ^19^, ^20^ is arbitrary. We study multiple covariates, age groups and settings, thereby increasing the possibility of chance findings. However, we have identified only one covariate that met the threshold.

There is a risk of bias associated with unadjusted IVE estimates.^42^ In our study, all estimates were minimally adjusted for age, sex, calendar time, and season. The finding that adjustment for additional variables did not lead to a meaningful impact on the IVE (with one exception) can be because the evaluated covariates are not true confounders, because of the low magnitude of the effect of true confounders, and/or because of the low prevalence of the variable in the population. Omitting variables with low prevalence only has a relatively small impact on IVE compared to more prevalent conditions with the same effect on the exposure and outcome.^43^

Although most estimates of the relative change in IVE were close to 1.0, indicating little to no impact of additional adjustment on the IVE estimates in the current dataset, variability was observed in the width of the CI. Multiple covariates analyzed in adults and older adults in the primary care setting and in children in the hospital setting had a CI that encompassed *a* > 10% relative change in the IVE, which represents an important side note when generalizing the findings to potential future IVE studies. In addition, in the primary care setting, the only stratification for which a confounder was found that led to *a* > 10% relative change in the IVE, the number of older adults was substantially lower than for the other age and setting stratifications.

A strength of the study is that the analysis is based on two seasons of data from a network of multiple European countries using harmonized data collection (through a core protocol and codebook). Furthermore, we started by building a theoretical framework to justify the selection of covariates in the confounder analysis and provided a reference point for confounding in future DRIVE and possibly other (network‐based) IVE studies.

Adequate confounder adjustment in vaccine monitoring studies is important to correctly interpret VE estimates for public health action and regulatory purposes. An efficient network collects data on a minimum set of variables that are directly relevant for the VE analyses. E‐values can be used to understand the magnitude an unmeasured confounder should have to cancel out the observed effect;^24^ however, no general rule can exist about small vs. large E‐values and thus the use of the E‐value should be considered as a complementary parameter only.^44^ Collecting additional variables may be of interest for descriptive purposes or for exploratory analyses but should not form a barrier to participation in a study network. Nevertheless, the impact of potential confounders on the observed effects should continue to be evaluated, especially if the context changes (e.g., relative vaccine effectiveness, COVID‐19 vaccines, etc.) or new data on potential confounders become available (e.g., on frailty).

## 5 CONCLUSION

The present study supports a parsimonious approach to confounder adjustment, limited to adjusting for age, sex, and calendar time, in network‐based TND IVE studies conducting analyses stratified by age groups and site and subsequently pooled by age and setting. For older adults in the primary care setting, additional adjustment for lung disease can be considered. The findings were reassuring as this parsimonious approach has been applied by the DRIVE since the 2019/2020 influenza season to produce brand‐specific IVE and report annual estimates to regulatory authorities. Practical implications are that necessitating fewer variables lowers the threshold for enrollment of sites in IVE studies and simplifies the pooling of data from different IVE studies or study networks, both of which are important to increase the sample size and geographic coverage (important for brand‐specific IVE assessment because of fragmented vaccine landscape) of IVE studies.

## CONFLICTS OF INTEREST

Anke Stuurman is an employee of P95. P95 holds/has held contracts with AstraZeneca, GSK, Sanofi, and Seqirus. Miriam Levi has no potential conflict of interest. Phillipe Beutels reports grants from the Innovative Medicines Initiative of the European Commission. He attended meetings of an advisory board on economic evaluations of vaccines convened by Pfizer in 2019, outside the submitted work. Hélène Bricout is a full‐time employee of Sanofi and holds shares in Sanofi. Alexandre Descamps reports consultation fees from Sanofi for advisory board meetings, outside the submitted work. Gael Dos Santos is an employee of the GSK group of companies and holds shares in the GSK group of companies as part of his annual remuneration. Ian McGovern is an employee of Seqirus and holds shares in Seqirus. Ainara Mira‐Iglesias has no potential conflict of interest. Jos Nauta is employed by Abbott, a company that produces influenza vaccines, and hold shares in Abbott. Laurence Torcel‐Pagnon is an employee of Sanofi and holds shares in Sanofi. Jorne Biccler is an employee of P95. P95 holds/has held contracts with AstraZeneca, GSK, Sanofi, and Seqirus.

## ETHICS STATEMENT

This study is a reanalysis of previously collected data. In the original studies, each local study was approved by national, regional, or institutional ethics committees, as appropriate. In the case of ISS, the study was submitted to the ethics committee for information, but approval was not required as the study is nested in the National Influenza Surveillance Scheme. Similarly, for the Finnish population‐based cohort study, an ethical evaluation was not mandatory; however, an evaluation from an institutional ethical review group was requested.

## AUTHOR CONTRIBUTIONS

**Anke L. Stuurman:** Conceptualization; investigation; methodology; project administration; validation; visualization; writing‐original draft. **Miriam Levi:** Conceptualization; investigation; methodology; project administration; validation; visualization; writing‐review and editing. **Philippe Beutels:** Conceptualization; investigation; methodology; project administration; validation; visualization; writing‐review and editing. **Hélène Bricout:** Conceptualization; investigation; methodology; project administration; validation; visualization; writing‐review and editing. **Alexandre Descamps:** Conceptualization; investigation; methodology; project administration; validation; visualization; writing‐review and editing. **Gael X Dos Santos:** Conceptualization; investigation; methodology; project administration; validation; visualization; writing‐review and editing. **Ian McGovern:** Conceptualization; investigation; methodology; project administration; validation; visualization; writing‐review and editing. **Ainara Mira‐Iglesias:** Conceptualization; investigation; methodology; project administration; validation; visualization; writing‐review and editing. **Jos Nauta:** Conceptualization; investigation; methodology; project administration; validation; visualization; writing‐review and editing. **Laurence Torcel‐Pagnon:** Conceptualization; investigation; methodology; project administration; validation; visualization; writing‐review and editing. **Jorne Biccler:** Conceptualization; data curation; formal analysis; investigation; methodology; project administration; validation; visualization; writing‐review and editing.

## CONSENT TO PARTICIPATE AND FOR PUBLICATION

This study is a reanalysis of previously collected data. In the original studies, all participants provided informed consent, when required by the ethics committee.

### PEER REVIEW

The peer review history for this article is available at https://publons.com/publon/10.1111/irv.13087.

## Supporting information


**Table S1a.** Characteristics of subjects in primary care setting with minimum data availability of age, sex, and calendar time.
**Table S1b.** Characteristics of subjects in hospital setting with minimum data availability of age, sex, and calendar time.
**Table S3a.** Subject counts and data availability for each variable by site each season, children at primary care sites.
**Table S3b.** Subject counts and data availability for each variable by site each season, children at hospital sites.
**Table S3c.** Subject counts and data availability for each variable by site each season, adults at primary care sites.
**Table S3d.** Subject counts and data availability for each variable by site each season, adults at hospital sites.
**Table S3e.** Subject counts and data availability for each variable by site each season, older adults at primary care sites.
**Table S3f.** Subject counts and data availability for each variable by site each season, older adults at hospital sites.
**Table S4a.** Propensity to be vaccinated among test‐negative controls in primary care setting. ORs are adjusted for age, sex, calendar time and season.
**Table S4b.** Propensity to be vaccinated among test‐negative controls in hospital setting. ORs are adjusted for age, sex, calendar time and season.
**Table S5a.** Propensity to test‐positive among vaccinated subjects in primary care setting. ORs are adjusted for age, sex, calendar time and season.
**Table S5b.** Propensity to test‐positive among vaccinated subjects in hospital. ORs are adjusted for age, sex, calendar time and season.
**Table S5c.** Propensity to test‐positive among unvaccinated subjects in primary care setting. ORs are adjusted for age, sex, calendar time and season.
**Table S5d.** Propensity to test‐positive among unvaccinated subjects in hospital setting. ORs are adjusted for age, sex, calendar time and season.
**Table S6a.** Absolute and relative changes in IVE estimate when adjusting for age, sex, calendar time and season (i.e. the reference model) compared to the model adjust for season only, by age group in the primary care setting.
**Table S6b.** Absolute and relative changes in IVE estimate when adjusting for age, sex, calendar time and season (i.e. the reference model) compared to the model adjust for season only, by age group in the hospital setting.
**Table S7.** Sensitivity analysis: relative changes in IVE estimate when adjusting for an additional covariate compared to the reference model adjusted for age, sex, calendar, season *and chronic conditions*; by age group and setting.
**Table S9.** E‐values for pooled VE point estimate and corresponding lower bound of 95%CI

## Data Availability

The data that support the findings of this study are available on request from the corresponding author. The data are not publicly available because of privacy or ethical restrictions.
