# Supplementary material for: Investigating confounding in network‐based test‐negative design influenza vaccine effectiveness studies—Experience from the DRIVE project
Source: Influenza Other Respir Viruses. 2022 Dec 22;17(1):e13087. doi: 10.1111/irv.13087 (PMC9835455; doi:10.1111/irv.13087)
Supplement: Supplementary file 1 — Table S1a. Characteristics of subjects in primary care setting with minimum data availability of age, sex, and calendar time. Table S1b. Characteristics of subjects in hospital setting with minimum data availability of age, sex, and calendar time. Table S3a. Subject counts and data availability for each variable by site each season, children at primary care sites. Table S3b. Subject counts and data availability for each variable by site each season, children at hospital sites. Table S3c. Subject counts and data availability for each variable by site each season, adults at primary care sites. Table S3d. Subject counts and data availability for each variable by site each season, adults at hospital sites. Table S3e. Subject counts and data availability for each variable by site each season, older adults at primary care sites. Table S3f. Subject counts and data availability for each variable by site each season, older adults at hospital sites. Table S4a. Propensity to be vaccinated among test‐negative controls in primary care setting. ORs are adjusted for age, sex, calendar time and season. Table S4b. Propensity to be vaccinated among test‐negative controls in hospital setting. ORs are adjusted for age, sex, calendar time and season. Table S5a. Propensity to test‐positive among vaccinated subjects in primary care setting. ORs are adjusted for age, sex, calendar time and season. Table S5b. Propensity to test‐positive among vaccinated subjects in hospital. ORs are adjusted for age, sex, calendar time and season. Table S5c. Propensity to test‐positive among unvaccinated subjects in primary care setting. ORs are adjusted for age, sex, calendar time and season. Table S5d. Propensity to test‐positive among unvaccinated subjects in hospital setting. ORs are adjusted for age, sex, calendar time and season. Table S6a. Absolute and relative changes in IVE estimate when adjusting for age, sex, calendar time and season (i.e. the reference model) compared to the model adjust for sea [file IRV-17-e13087-s001.docx]

SUPPLEMENT 1: SUBJECT CHARACTERISTICS

**Table S1a. Characteristics of subjects in primary care setting with minimum data availability of age, sex, and calendar time.**

| PRIMARY CARE | | Children | | | | Adults | | | | Older adults | | | |
| --- | --- | --- | --- | --- | --- | --- | --- | --- | --- | --- | --- | --- | --- |
|  |  | Vaccinated | Vaccinated | Unvaccinated | Unvaccinated | Vaccinated | Vaccinated | Unvaccinated | Unvaccinated | Vaccinated | Vaccinated | Unvaccinated | Unvaccinated |
| covariate | value | Controls | Cases | Controls | Cases | Controls | Cases | Controls | Cases | Controls | Cases | Controls | Cases |
| chronic | Absent | 196 (84%) | 104 (76%) | 1770 (95%) | 2008 (94%) | 121 (41%) | 58 (48%) | 1804 (78%) | 1236 (81%) | 57 (16%) | 27 (22%) | 71 (34%) | 35 (41%) |
| chronic | Present | 38 (16%) | 33 (24%) | 99 (5%) | 120 (6%) | 175 (59%) | 63 (52%) | 509 (22%) | 290 (19%) | 291 (84%) | 97 (78%) | 140 (66%) | 50 (59%) |
| chronic2 | Absent | - | - | 953 (100%) | 1114 (100%) | 159 (87%) | 51 (91%) | 1269 (96%) | 791 (97%) | 118 (53%) | 36 (64%) | 111 (79%) | 35 (71%) |
| chronic2 | Present | - | - | 1 (0%) | NA (NA%) | 23 (13%) | 5 (9%) | 51 (4%) | 23 (3%) | 106 (47%) | 20 (36%) | 30 (21%) | 14 (29%) |
| chronic3 | Absent | - | - | - | - | 176 (97%) | 55 (98%) | 1310 (99%) | 810 (100%) | 177 (79%) | 43 (77%) | 128 (91%) | 45 (92%) |
| chronic3 | Present | - | - | - | - | 6 (3%) | 1 (2%) | 10 (1%) | 4 (0%) | 47 (21%) | 13 (23%) | 13 (9%) | 4 (8%) |
| cardiovasc | Absent | 113 (97%) | 47 (98%) | 679 (100%) | 694 (100%) | 107 (82%) | 26 (74%) | 817 (91%) | 394 (93%) | 68 (34%) | 12 (26%) | 64 (62%) | 12 (39%) |
| cardiovasc | Present | 3 (3%) | 1 (2%) | 2 (0%) | 3 (0%) | 24 (18%) | 9 (26%) | 85 (9%) | 30 (7%) | 130 (66%) | 35 (74%) | 39 (38%) | 19 (61%) |
| cancer | Absent | - | - | 698 (100%) | 720 (100%) | 142 (95%) | 35 (92%) | 948 (98%) | 426 (99%) | 181 (87%) | 41 (87%) | 90 (87%) | 21 (81%) |
| cancer | Present | - | - | 2 (0%) | NA (NA%) | 7 (5%) | 3 (8%) | 15 (2%) | 6 (1%) | 27 (13%) | 6 (13%) | 14 (13%) | 5 (19%) |
| diabetes | Absent | - | - | - | - | 141 (87%) | 38 (95%) | 976 (97%) | 442 (97%) | 169 (76%) | 42 (82%) | 91 (81%) | 20 (77%) |
| diabetes | Present | - | - | - | - | 21 (13%) | 2 (5%) | 30 (3%) | 13 (3%) | 52 (24%) | 9 (18%) | 22 (19%) | 6 (23%) |
| lungdis | Absent | 128 (97%) | 49 (96%) | 710 (97%) | 717 (98%) | 121 (75%) | 30 (77%) | 923 (91%) | 424 (94%) | 149 (67%) | 39 (76%) | 87 (79%) | 22 (85%) |
| lungdis | Present | 4 (3%) | 2 (4%) | 19 (3%) | 14 (2%) | 41 (25%) | 9 (23%) | 88 (9%) | 27 (6%) | 72 (33%) | 12 (24%) | 23 (21%) | 4 (15%) |
| obesity | Absent | - | - | 889 (99%) | 1057 (98%) | 134 (89%) | 50 (96%) | 1072 (89%) | 688 (89%) | 176 (89%) | 47 (92%) | 120 (92%) | 41 (84%) |
| obesity | Present | - | - | 11 (1%) | 19 (2%) | 17 (11%) | 2 (4%) | 127 (11%) | 83 (11%) | 21 (11%) | 4 (8%) | 11 (8%) | 8 (16%) |
| rendisease | Absent | - | - | 726 (100%) | 729 (100%) | 159 (99%) | 39 (98%) | 999 (100%) | 444 (100%) | 199 (90%) | 45 (90%) | 104 (95%) | 21 (95%) |
| rendisease | Present | - | - | NA (NA%) | 1 (0%) | 2 (1%) | 1 (2%) | 4 (0%) | 1 (0%) | 21 (10%) | 5 (10%) | 5 (5%) | 1 (5%) |
| nhosp | 0 | 144 (94%) | 77 (95%) | 1162 (98%) | 1355 (98%) | 213 (96%) | 93 (97%) | 1614 (97%) | 916 (98%) | 272 (88%) | 101 (93%) | 146 (91%) | 50 (93%) |
| nhosp | 1 to 2 | 8 (5%) | 4 (5%) | 19 (2%) | 25 (2%) | 10 (4%) | 3 (3%) | 40 (2%) | 19 (2%) | 33 (11%) | 8 (7%) | 13 (8%) | 4 (7%) |
| nhosp | more than 2 | 2 (1%) | NA (NA%) | 7 (1%) | NA (NA%) | - | - | 4 (0%) | 2 (0%) | 3 (1%) | NA (NA%) | 1 (1%) | NA (NA%) |
| gpvisit | 0 | 10 (7%) | 9 (9%) | 177 (15%) | 201 (15%) | 20 (12%) | 17 (23%) | 355 (26%) | 244 (30%) | 12 (6%) | 4 (5%) | 11 (10%) | 3 (8%) |
| gpvisit | 1 to 5 | 102 (71%) | 80 (77%) | 820 (70%) | 1022 (74%) | 108 (66%) | 47 (63%) | 898 (67%) | 531 (65%) | 123 (58%) | 54 (67%) | 67 (62%) | 24 (67%) |
| gpvisit | more than 5 | 31 (22%) | 15 (14%) | 179 (15%) | 155 (11%) | 36 (22%) | 11 (15%) | 95 (7%) | 45 (5%) | 77 (36%) | 23 (28%) | 30 (28%) | 9 (25%) |

Chronic: presence of at least one chronic condition; chronic2: presence of at least two chronic conditions; chronic3: presence of at least three chronic conditions; cardiovasc: cardiovascular disease;
lungdis: lung disease; rendisease: renal disease; nhosp: number of hospitalizations in previous year; gpvisit: number of general practice visits in previous year.

**Table S1b. Characteristics of subjects in hospital setting with minimum data availability of age, sex, and calendar time.**

| HOSPITAL |  | Children | | | | Adults | | | | Older adults | | | |
| --- | --- | --- | --- | --- | --- | --- | --- | --- | --- | --- | --- | --- | --- |
| covariate | value | Vaccinated | Vaccinated | Unvaccinated | Unvaccinated | Vaccinated | Vaccinated | Unvaccinated | Unvaccinated | Vaccinated | Vaccinated | Unvaccinated | Unvaccinated |
|  |  | Controls | Cases | Controls | Cases | Controls | Cases | Controls | Cases | Controls | Cases | Controls | Cases |
| chronic | Absent | 47 (52%) | 17 (45%) | 1464 (85%) | 951 (84%) | 46 (14%) | 25 (25%) | 439 (39%) | 216 (36%) | 62 (4%) | 19 (5%) | 122 (10%) | 32 (7%) |
| chronic | Present | 44 (48%) | 21 (55%) | 259 (15%) | 184 (16%) | 272 (86%) | 75 (75%) | 693 (61%) | 386 (64%) | 1703 (96%) | 362 (95%) | 1117 (90%) | 450 (93%) |
| chronic2 | Absent | 75 (95%) | 29 (97%) | 1640 (99%) | 1055 (99%) | 145 (51%) | 40 (53%) | 737 (70%) | 334 (66%) | 438 (26%) | 91 (31%) | 432 (37%) | 147 (38%) |
| chronic2 | Present | 4 (5%) | 1 (3%) | 21 (1%) | 14 (1%) | 142 (49%) | 36 (47%) | 314 (30%) | 170 (34%) | 1220 (74%) | 198 (69%) | 726 (63%) | 244 (62%) |
| chronic3 | Absent | 78 (99%) | 30 (100%) | 1656 (100%) | 1067 (100%) | 201 (70%) | 57 (75%) | 888 (84%) | 418 (83%) | 922 (56%) | 181 (63%) | 738 (64%) | 270 (69%) |
| chronic3 | Present | 1 (1%) | NA (NA%) | 5 (0%) | 2 (0%) | 86 (30%) | 19 (25%) | 163 (16%) | 86 (17%) | 736 (44%) | 108 (37%) | 420 (36%) | 121 (31%) |
| cardiovasc | Absent | 70 (89%) | 29 (97%) | 1629 (98%) | 1046 (98%) | 213 (74%) | 59 (78%) | 872 (83%) | 390 (77%) | 603 (36%) | 119 (41%) | 499 (43%) | 157 (40%) |
| cardiovasc | Present | 9 (11%) | 1 (3%) | 32 (2%) | 23 (2%) | 74 (26%) | 17 (22%) | 179 (17%) | 114 (23%) | 1054 (64%) | 170 (59%) | 658 (57%) | 234 (60%) |
| cancer | Absent | - | - | 1652 (99%) | 1065 (100%) | 254 (89%) | 71 (93%) | 968 (92%) | 472 (94%) | 1413 (85%) | 253 (88%) | 960 (83%) | 342 (87%) |
| cancer | Present | - | - | 9 (1%) | 4 (0%) | 33 (11%) | 5 (7%) | 83 (8%) | 32 (6%) | 245 (15%) | 36 (12%) | 197 (17%) | 49 (13%) |
| diabetes | Absent | 78 (99%) | 30 (100%) | 1656 (100%) | 1060 (99%) | 229 (80%) | 63 (83%) | 946 (90%) | 440 (87%) | 1067 (64%) | 189 (65%) | 795 (69%) | 297 (76%) |
| diabetes | Present | 1 (1%) | NA (NA%) | 5 (0%) | 9 (1%) | 58 (20%) | 13 (17%) | 105 (10%) | 64 (13%) | 591 (36%) | 100 (35%) | 363 (31%) | 94 (24%) |
| lungdis | Absent | 67 (85%) | 27 (90%) | 1604 (97%) | 1036 (97%) | 175 (61%) | 49 (65%) | 832 (79%) | 395 (78%) | 848 (51%) | 153 (53%) | 754 (65%) | 245 (63%) |
| lungdis | Present | 12 (15%) | 3 (10%) | 57 (3%) | 33 (3%) | 111 (39%) | 26 (35%) | 219 (21%) | 109 (22%) | 809 (49%) | 136 (47%) | 404 (35%) | 146 (37%) |
| obesity | Absent | 75 (95%) | 30 (100%) | 1568 (94%) | 1056 (99%) | 217 (76%) | 61 (80%) | 880 (84%) | 420 (83%) | 1306 (79%) | 231 (80%) | 946 (82%) | 325 (83%) |
| obesity | Present | 4 (5%) | NA (NA%) | 92 (6%) | 13 (1%) | 70 (24%) | 15 (20%) | 170 (16%) | 84 (17%) | 352 (21%) | 58 (20%) | 212 (18%) | 66 (17%) |
| rendisease | Absent | 77 (97%) | 27 (90%) | 1647 (99%) | 1053 (99%) | 257 (90%) | 69 (91%) | 994 (95%) | 468 (93%) | 1328 (80%) | 243 (84%) | 946 (82%) | 338 (86%) |
| rendisease | Present | 2 (3%) | 3 (10%) | 14 (1%) | 16 (1%) | 30 (10%) | 7 (9%) | 57 (5%) | 36 (7%) | 330 (20%) | 46 (16%) | 212 (18%) | 53 (14%) |
| nhosp | 0 | 36 (51%) | 21 (75%) | 467 (60%) | 416 (61%) | 171 (59%) | 46 (50%) | 573 (64%) | 310 (65%) | 944 (57%) | 219 (67%) | 599 (57%) | 222 (56%) |
| nhosp | 1 to 2 | 27 (39%) | 5 (18%) | 249 (32%) | 203 (30%) | 86 (30%) | 32 (35%) | 236 (26%) | 132 (28%) | 548 (33%) | 86 (26%) | 360 (34%) | 141 (36%) |
| nhosp | more than 2 | 7 (10%) | 2 (7%) | 57 (7%) | 59 (9%) | 31 (11%) | 14 (15%) | 82 (9%) | 36 (8%) | 168 (10%) | 21 (6%) | 90 (9%) | 33 (8%) |
| gpvisit | 0 | 10 (18%) | 5 (45%) | 119 (15%) | 120 (22%) | 32 (22%) | 9 (21%) | 223 (39%) | 84 (31%) | 121 (11%) | 34 (19%) | 138 (20%) | 57 (24%) |
| gpvisit | 1 to 5 | 16 (29%) | 3 (27%) | 446 (55%) | 331 (62%) | 28 (19%) | 16 (38%) | 158 (27%) | 109 (40%) | 194 (18%) | 29 (16%) | 188 (27%) | 75 (32%) |
| gpvisit | more than 5 | 9 (16%) | 3 (27%) | 113 (14%) | 68 (13%) | 6 (4%) | 2 (5%) | 22 (4%) | 25 (9%) | 72 (7%) | 16 (9%) | 40 (6%) | 32 (13%) |

Chronic: presence of at least one chronic condition; chronic2: presence of at least two chronic conditions; chronic3: presence of at least three chronic conditions; cardiovasc: cardiovascular disease; lungdis:
lung disease; rendisease: renal disease; nhosp: number of hospitalizations in previous year; gpvisit: number of general practice visits in previous year.

SUPPLEMENT 2: DEFINITIONS OF CHRONIC CONDITIONS

| Condition | Definition |
| --- | --- |
| Chronic liver disease | Any of the following dg codes (ICD-10)*: B18, K70-74, K75.0-75.1, K75.3-75.9, K76-77  INCLUDING: Alcoholic liver disease, Toxic liver disease, Hepatic failure, Chronic hepatitis (viral & other), Fibrosis and cirrhosis of liver, Other inflammatory liver diseases, Other diseases of liver  EXCLUDING: Clinically insignificant liver cysts |
| Diabetes | Any of the following dg codes (ICD-10)*: E10-E14, O24  INCLUDING: Any form of diabetes, including sequelae & DM in pregnancy |
| Cardiovascular diseases | Any of the following dg codes (ICD-10)*: A52.0, B37.6, I01-02, I05-09, I11.0, I13.0, I13.2, I20-25, I26-28, I30-43, I44-46, I48, I49.0, I49.5, I50-52, I70-71, Q20-Q28  INCLUDING: all conditions of heart & large vessels that are chronic or likely to have chronic sequelae. Cardiovascular syphilis, endo-, myo- and pericarditis, rheumatic fever, chronic rheumatic heart diseases, congenital malformations, hypertensive (renal) diseases with heart failure, ischaemic heart diseases, diseases of pulmonary circulation, atherosclerosis, cardiomyopathies, most conduction disorders, heart failure, aortic aneurysms & dissecation, other heart diseases and their complications.  EXCLUDING: uncomplicated hypertension, previous uncomplicated pulmonary embolism (with no lasting cardiac insufficiency), paroxysmal tachycardias, most cases of premature depolarization. |
| Cancer | Any of the following dg codes (ICD-10)*: C00-97, D37-48, Z85, Z92.3, Z92.6.  INCLUDING: All malignant neoplasms (both solid and haematologic) with potential to metastasize, either in treatment, active followup, or <5 years post curative treatment.  EXCLUDING: Benign & in situ neoplasms. Basal cell carcinomas. Any cancer previously treated with curative intent & in complete remission for ≥5 years. |
| Immuno-deficiency or organ transplant | Any of the following dg codes (ICD-10)*: B20-B24, D80–84, D89, Z94  INCLUDING: HIV infections, immunodeficiencies & organ transplants. or iatrogenic: ≥2 week systemic treatment, in the 3 months preceding symptom onset, with any of the following: corticosteroid (≥20 mg prednisolone daily or equivalent), ciclosporin, tacrolimus, mycophenolate, methotrexate, azathioprine, TNF-α blockers and other biological or cytostatic drugs with immunosuppressive effect  EXCLUDING: Disorders of the immune system which do not lead to immunosuppression (e.g. some autoimmune conditions). |

Source: Rizzo C, Alfonsi V, Bollaerts K, Riera M, Stuurman A, Turunen T. D7.1 Core protocol for type/brand-specific influenza vaccine effectiveness studies (test-negative design studies). 2018. https://www.drive-eu.org/wp-content/uploads/2018/12/DRIVE_D7.1_Core-protocol-for-test-negative-design-studies_1.1.pdf Accessed 22 April 2022.

SUPPLEMENT 3: DATA AVAILABILITY

BIVE: Italian Hospital Network; CIRI-IT: Interuniversity Research Center on Influenza and other Transmissible Infections; FISABIO: Foundation for the Promotion of Health and Biomedical Research of the Valencia Region; GP: General practice; GTPUH: Germans Trias I Pujol University Hospital; HUS: Helsinki University Hospital, Jorvi Hospital; INSERM/I-REIVAC: Innovative Clinical Research Network In Vaccinology; LPUH: La Paz University Hospital; NIID: National Institute for Infectious Diseases "Prof. Dr. Matei Balş"; ISS: Italian National Institute of Health; MUV: Medical University Vienna; RCGP: Royal College of General Practitioners Research and Surveillance Centre; VHUH: Vall d'Hebron University Hospital

**Table S3a. Subject counts and data availability for each variable by site each season, children at primary care sites.**

**Table S3b. Subject counts and data availability for each variable by site each season, children at hospital sites.**

**Table S3c. Subject counts and data availability for each variable by site each season, adults at primary care sites.**

**Table S3d. Subject counts and data availability for each variable by site each season, adults at hospital sites.**

**Table S3e. Subject counts and data availability for each variable by site each season, older adults at primary care sites.**

**Table S3f. Subject counts and data availability for each variable by site each season, older adults at hospital sites.**

SUPPLEMENT 4: PREDICTORS OF VACCINATION

**Table S4a. Propensity to be vaccinated among test-negative controls in primary care setting. ORs are adjusted for age, sex, calendar time and season.**

|  |  | | Children | | | | Adults | | | | Older adults | | |
| --- | --- | --- | --- | --- | --- | --- | --- | --- | --- | --- | --- | --- | --- |
| covariate | value | Unvaccinated, n(%) | | Vaccinated, n(%) | OR  (95%CI) | Unvaccinated, n(%) | | Vaccinated, n(%) | OR  (95%CI) | Unvaccinated, n(%) | | Vaccinated, n(%) | OR  (95%CI) |
| chronic | Absent | 1770 (95%) | | 196 (84%) | ref | 1804 (78%) | | 121 (41%) | ref | 71 (34%) | | 57 (16%) | ref |
| chronic | Present | 99 (5%) | | 38 (16%) | 4.5 (2.9; 7.0) | 509 (22%) | | 175 (59%) | 3.8 (2.9; 5.0) | 140 (66%) | | 291 (84%) | 2.3 (1.5; 3.7) |
| chronic2 | Absent | 953 (100%) | | 155 (100%) | ref | 1269 (96%) | | 159 (87%) | ref | 111 (79%) | | 118 (53%) | ref |
| chronic2 | Present | 1 (0%) | | NA (NA%) | 0 (0; Inf) | 51 (4%) | | 23 (13%) | 2.0 (1.1; 3.6) | 30 (21%) | | 106 (47%) | 2.9 (1.6; 5.0) |
| chronic3 | Absent | - | | - | - | 1310 (99%) | | 176 (97%) | ref | 128 (91%) | | 177 (79%) | ref |
| chronic3 | Present | - | | - | - | 10 (1%) | | 6 (3%) | 2.9 (0.9; 8.6) | 13 (9%) | | 47 (21%) | 2.1 (1.0; 4.4) |
| cancer | Absent | 698 (100%) | | 129 (100%) | ref | 948 (98%) | | 142 (95%) | ref | 90 (87%) | | 181 (87%) | ref |
| cancer | Present | 2 (0%) | | NA (NA%) | 0 (0; Inf) | 15 (2%) | | 7 (5%) | 1.7 (0.6; 4.7) | 14 (13%) | | 27 (13%) | 1.3 (0.6; 3.1) |
| cardiovasc | Absent | 679 (100%) | | 113 (97%) | ref | 817 (91%) | | 107 (82%) | ref | 64 (62%) | | 68 (34%) | ref |
| cardiovasc | Present | 2 (0%) | | 3 (3%) | 10.5 (1.6; 70.4) | 85 (9%) | | 24 (18%) | 1.2 (0.7; 2.1) | 39 (38%) | | 130 (66%) | 2.7 (1.5; 4.8) |
| diabetes | Absent | - | | - | - | 976 (97%) | | 141 (87%) | ref | 91 (81%) | | 169 (76%) | ref |
| diabetes | Present | - | | - | - | 30 (3%) | | 21 (13%) | 3.2 (1.7; 6.1) | 22 (19%) | | 52 (24%) | 1.5 (0.8; 3.0) |
| lungdis | Absent | 710 (97%) | | 128 (97%) | ref | 923 (91%) | | 121 (75%) | ref | 87 (79%) | | 149 (67%) | ref |
| lungdis | Present | 19 (3%) | | 4 (3%) | 1.5 (0.5; 4.8) | 88 (9%) | | 41 (25%) | 3.9 (2.5; 6.2) | 23 (21%) | | 72 (33%) | 2.0 (1.1; 3.7) |
| obesity | Absent | 889 (99%) | | 138 (100%) | ref | 1072 (89%) | | 134 (89%) | ref | 120 (92%) | | 176 (89%) | ref |
| obesity | Present | 11 (1%) | | NA (NA%) | 0 (0; Inf) | 127 (11%) | | 17 (11%) | 1.4 (0.8; 2.6) | 11 (8%) | | 21 (11%) | 4.7 (1.4; 15.5) |
| rendisease | Absent | - | | - | - | 999 (100%) | | 159 (99%) | ref | 104 (95%) | | 199 (90%) | ref |
| rendisease | Present | - | | - | - | 4 (0%) | | 2 (1%) | 2.2 (0.3; 14.4) | 5 (5%) | | 21 (10%) | 2.1 (0.6; 6.8) |
| nhosp | 0 | 1162 (98%) | | 144 (94%) | ref | 1614 (97%) | | 213 (96%) | ref | 146 (91%) | | 272 (88%) | ref |
| nhosp | 1 to 2 | 19 (2%) | | 8 (5%) | 5.1 (2.1; 12.5) | 40 (2%) | | 10 (4%) | 1.3 (0.6; 2.8) | 13 (8%) | | 33 (11%) | 1.4 (0.7; 3.0) |
| nhosp | more than 2 | 7 (1%) | | 2 (1%) | 3.3 (0.6; 18.0) | 4 (0%) | | NA (NA%) | 0 (0; Inf) | 1 (1%) | | 3 (1%) | 1.2 (0.1; 11.9) |
| gpvisit | 0 | 177 (15%) | | 10 (7%) | ref | 355 (26%) | | 20 (12%) | ref | 11 (10%) | | 12 (6%) | ref |
| gpvisit | 1 to 5 | 820 (70%) | | 102 (71%) | 2.8 (1.4; 5.6) | 898 (67%) | | 108 (66%) | 1.7 (1.0; 2.8) | 67 (62%) | | 123 (58%) | 1.9 (0.8; 4.8) |
| gpvisit | more than 5 | 179 (15%) | | 31 (22%) | 4.3 (1.9; 9.5) | 95 (7%) | | 36 (22%) | 3.9 (2.1; 7.3) | 30 (28%) | | 77 (36%) | 2.2 (0.8; 5.8) |

Chronic: presence of at least one chronic condition; chronic2: presence of at least two chronic conditions; chronic3: presence of at least three chronic conditions; cardiovasc: cardiovascular disease; lungdis: lung disease; rendisease: renal disease; nhosp: number of hospitalizations in previous year; gpvisit: number of general practice visits in previous year.

**Table S4b. Propensity to be vaccinated among test-negative controls in hospital setting. ORs are adjusted for age, sex, calendar time and season.**

|  |  | Children | | | Adults | | | Older adults | | |
| --- | --- | --- | --- | --- | --- | --- | --- | --- | --- | --- |
| covariate | value | Unvaccinated, n(%) | Vaccinated, n(%) | OR  (95%CI) | Unvaccinated, n(%) | Vaccinated, n(%) | OR  (95%CI) | Unvaccinated, n(%) | Vaccinated, n(%) | OR  (95%CI) |
| chronic | Absent | 1464 (85%) | 47 (52%) | ref | 439 (39%) | 46 (14%) | ref | 122 (10%) | 62 (4%) | ref |
| chronic | Present | 259 (15%) | 44 (48%) | 3.8 (2.3; 6.3) | 693 (61%) | 272 (86%) | 2.5 (1.8; 3.7) | 1117 (90%) | 1703 (96%) | 2.6 (1.9; 3.7) |
| chronic2 | Absent | 1640 (99%) | 75 (95%) | ref | 737 (70%) | 145 (51%) | ref | 432 (37%) | 438 (26%) | ref |
| chronic2 | Present | 21 (1%) | 4 (5%) | 2.4 (0.7; 7.6) | 314 (30%) | 142 (49%) | 1.6 (1.2; 2.2) | 726 (63%) | 1220 (74%) | 1.5 (1.3; 1.8) |
| chronic3 | Absent | 1656 (100%) | 78 (99%) | ref | 888 (84%) | 201 (70%) | ref | 738 (64%) | 922 (56%) | ref |
| chronic3 | Present | 5 (0%) | 1 (1%) | 3.3 (0.3; 34.3) | 163 (16%) | 86 (30%) | 1.6 (1.1; 2.2) | 420 (36%) | 736 (44%) | 1.3 (1.1; 1.5) |
| cancer | Absent | 1652 (99%) | 79 (100%) | ref | 968 (92%) | 254 (89%) | ref | 960 (83%) | 1413 (85%) | ref |
| cancer | Present | 9 (1%) | NA (NA%) | 0 (0; Inf) | 83 (8%) | 33 (11%) | 1.2 (0.8; 2.0) | 197 (17%) | 245 (15%) | 0.9 (0.7; 1.1) |
| cardiovasc | Absent | 1629 (98%) | 70 (89%) | ref | 872 (83%) | 213 (74%) | ref | 499 (43%) | 603 (36%) | ref |
| cardiovasc | Present | 32 (2%) | 9 (11%) | 6.3 (2.8; 14.3) | 179 (17%) | 74 (26%) | 1.6 (1.1; 2.2) | 658 (57%) | 1054 (64%) | 1.4 (1.2; 1.6) |
| diabetes | Absent | 1656 (100%) | 78 (99%) | ref | 946 (90%) | 229 (80%) | ref | 795 (69%) | 1067 (64%) | ref |
| diabetes | Present | 5 (0%) | 1 (1%) | 5.9 (0.6; 53.8) | 105 (10%) | 58 (20%) | 1.7 (1.1; 2.5) | 363 (31%) | 591 (36%) | 1.2 (1.0; 1.4) |
| lungdis | Absent | 1604 (97%) | 67 (85%) | ref | 832 (79%) | 175 (61%) | ref | 754 (65%) | 848 (51%) | ref |
| lungdis | Present | 57 (3%) | 12 (15%) | 3.3 (1.6; 6.8) | 219 (21%) | 111 (39%) | 1.6 (1.2; 2.2) | 404 (35%) | 809 (49%) | 1.7 (1.4; 2.0) |
| obesity | Absent | 1568 (94%) | 75 (95%) | ref | 880 (84%) | 217 (76%) | ref | 946 (82%) | 1306 (79%) | ref |
| obesity | Present | 92 (6%) | 4 (5%) | 0.2 (0.1; 0.6) | 170 (16%) | 70 (24%) | 1.1 (0.8; 1.6) | 212 (18%) | 352 (21%) | 1.0 (0.8; 1.2) |
| rendisease | Absent | 1647 (99%) | 77 (97%) | ref | 994 (95%) | 257 (90%) | ref | 946 (82%) | 1328 (80%) | ref |
| rendisease | Present | 14 (1%) | 2 (3%) | 2.2 (0.4; 10.8) | 57 (5%) | 30 (10%) | 1.7 (1.1; 2.9) | 212 (18%) | 330 (20%) | 1.0 (0.8; 1.2) |
| nhosp | 0 | 467 (60%) | 36 (51%) | ref | 573 (64%) | 171 (59%) | ref | 599 (57%) | 944 (57%) | ref |
| nhosp | 1 to 2 | 249 (32%) | 27 (39%) | 1.7 (1.0; 3.0) | 236 (26%) | 86 (30%) | 1.2 (0.8; 1.6) | 360 (34%) | 548 (33%) | 1.0 (0.9; 1.2) |
| nhosp | more than 2 | 57 (7%) | 7 (10%) | 1.5 (0.6; 4.0) | 82 (9%) | 31 (11%) | 1.2 (0.8; 2.0) | 90 (9%) | 168 (10%) | 1.2 (0.9; 1.6) |
| gpvisit | 0 | 119 (15%) | 10 (18%) | ref | 223 (39%) | 32 (22%) | ref | 138 (20%) | 121 (11%) | ref |
| gpvisit | 1 to 5 | 446 (55%) | 16 (29%) | 0.6 (0.2; 1.6) | 158 (27%) | 28 (19%) | 1.3 (0.7; 2.5) | 188 (27%) | 194 (18%) | 1.8 (1.2; 2.7) |
| gpvisit | more than 5 | 113 (14%) | 9 (16%) | 2 (0.6; 6.1) | 22 (4%) | 6 (4%) | 2.7 (0.8; 8.9) | 40 (6%) | 72 (7%) | 2.8 (1.6; 4.9) |

Chronic: presence of at least one chronic condition; chronic2: presence of at least two chronic conditions; chronic3: presence of at least three chronic conditions; cardiovasc: cardiovascular disease; lungdis: lung disease; rendisease: renal disease; nhosp: number of hospitalizations in previous year; gpvisit: number of general practice visits in previous year.

SUPPLEMENT 5: PREDICTORS OF THE OUTCOME

**Table S5a. Propensity to test-positive among vaccinated subjects in primary care setting. ORs are adjusted for age, sex, calendar time and season.**

| Primary care |  | Children - vaccinated | | | Adults - vaccinated | | | Older adults - vaccinated | | | |  |
| --- | --- | --- | --- | --- | --- | --- | --- | --- | --- | --- | --- | --- |
| covariate | value | Control  n(%) | Cases  n(%) | OR  (95%CI) | Controls  n(%) | Cases  n(%) | OR  (95%CI) | | Controls  n(%) | Cases  n(%) | OR  (95%CI) | |
| chronic | Absent | 196 (84%) | 104 (76%) | ref | 121 (41%) | 58 (48%) | ref | | 57 (16%) | 27 (22%) | ref | |
| chronic | Present | 38 (16%) | 33 (24%) | 1.2 (0.6; 2.2) | 175 (59%) | 63 (52%) | 1.0 (0.6; 1.6) | | 291 (84%) | 97 (78%) | 0.7 (0.4; 1.3) | |
| chronic2 | Absent | - | - | - | 159 (87%) | 51 (91%) | ref | | 118 (53%) | 36 (64%) | ref | |
| chronic2 | Present | - | - | - | 23 (13%) | 5 (9%) | 0.9 (0.3; 2.6) | | 106 (47%) | 20 (36%) | 0.6 (0.3; 1.2) | |
| chronic3 | Absent | - | - | - | 176 (97%) | 55 (98%) | ref | | 177 (79%) | 43 (77%) | ref | |
| chronic3 | Present | - | - | - | 6 (3%) | 1 (2%) | 0.6 (0.1; 6.1) | | 47 (21%) | 13 (23%) | 1.2 (0.5; 2.5) | |
| cancer | Absent | - | - | - | 142 (95%) | 35 (92%) | ref | | 181 (87%) | 41 (87%) | ref | |
| cancer | Present | - | - | - | 7 (5%) | 3 (8%) | 3.5 (0.7; 18.7) | | 27 (13%) | 6 (13%) | 1.3 (0.4; 3.8) | |
| cardiovasc | Absent | 13 (97%) | 47 (98%) | ref | 107 (82%) | 26 (74%) | ref | | 68 (34%) | 12 (26%) | ref | |
| cardiovasc | Present | (3%) | 1 (2%) | 2 (0.1; 26.6) | 24 (18%) | 9 (26%) | 2.3 (0.8; 6.7) | | 130 (66%) | 35 (74%) | 1.7 (0.8; 3.9) | |
| diabetes | Absent | - | - | - | 141 (87%) | 38 (95%) | ref | | 169 (76%) | 42 (82%) | ref | |
| diabetes | Present | - | - | - | 21 (13%) | 2 (5%) | 0.5 (0.1; 2.6) | | 52 (24%) | 9 (18%) | 0.6 (0.3; 1.6) | |
| lungdis | Absent | 128 (97%) | 49 (96%) | ref | 121 (75%) | 30 (77%) | ref | | 149 (67%) | 39 (76%) | ref | |
| lungdis | Present | 4 (3%) | 2 (4%) | 1.1 (0.1; 8.3) | 41 (25%) | 9 (23%) | 0.7 (0.3; 1.6) | | 72 (33%) | 12 (24%) | 0.5 (0.2; 1.1) | |
| obesity | Absent | - | - | - | 134 (89%) | 50 (96%) | ref | | 176 (89%) | 47 (92%) | ref | |
| obesity | Present | - | - | - | 17 (11%) | 2 (4%) | 0.3 (0.1; 1.2) | | 21 (11%) | 4 (8%) | 0.4 (0.1; 1.5) | |
| rendisease | Absent | - | - | - | 159 (99%) | 39 (98%) | ref | | 199 (90%) | 45 (90%) | ref | |
| rendisease | Present | - | - | - | 2 (1%) | 1 (2%) | 3.4 (0.2; 49.1) | | 21 (10%) | 5 (10%) | 1.2 (0.4; 3.9) | |
| nhosp | 0 | 144 (94%) | 77 (95%) | ref | 213 (96%) | 93 (97%) | ref | | 272 (88%) | 101 (93%) | ref | |
| nhosp | 1 to 2 | 8 (5%) | 4 (5%) | 0.6 (0.2; 2.4) | 10 (4%) | 3 (3%) | 0.8 (0.2; 3.3) | | 33 (11%) | 8 (7%) | 0.7 (0.3; 1.6) | |
| nhosp | more than 2 | 2 (1%) | NA (NA%) | 0 (0; Inf) |  |  |  | | 3 (1%) | NA (NA%) | 0 (0; Inf) | |
| gpvisit | 0 | 10 (7%) | 9 (9%) | ref | 20 (12%) | 17 (23%) | ref | | 12 (6%) | 4 (5%) | ref | |
| gpvisit | 1 to 5 | 102 (71%) | 80 (77%) | 0.9 (0.3; 2.8) | 108 (66%) | 47 (63%) | 0.3 (0.1; 0.8) | | 123 (58%) | 54 (67%) | 1.7 (0.4; 7.1) | |
| gpvisit | more than 5 | 31 (22%) | 15 (14%) | 0.8 (0.2; 2.7) | 36 (22%) | 11 (15%) | 0.2 (0.1; 0.7) | | 77 (36%) | 23 (28%) | 1.1 (0.3; 4.7) | |

Chronic: presence of at least one chronic condition; chronic2: presence of at least two chronic conditions; chronic3: presence of at least three chronic conditions; cardiovasc: cardiovascular disease; lungdis: lung disease; rendisease: renal disease; nhosp: number of hospitalizations in previous year; gpvisit: number of general practice visits in previous year.

**Table S5b. Propensity to test-positive among vaccinated subjects in hospital. ORs are adjusted for age, sex, calendar time and season.**

| Hospital |  | Children - vaccinated | | | Adults - vaccinated | | | Older adults - vaccinated | | | |
| --- | --- | --- | --- | --- | --- | --- | --- | --- | --- | --- | --- |
| covariate | value | Controls  n(%) | Cases  n(%) | OR  (95%CI) | Controls  n(%) | Cases  n(%) | OR  (95%CI) | Controls  n(%) | Cases  n(%) | OR  (95%CI) |  |
| chronic | Absent | 47 (52%) | 17 (45%) | ref | 46 (14%) | 25 (25%) | ref | 62 (4%) | 19 (5%) | ref |  |
| chronic | Present | 44 (48%) | 21 (55%) | 2.3 (0.8; 6.6) | 272 (86%) | 75 (75%) | 1.0 (0.5; 2.0) | 1703 (96%) | 362 (95%) | 0.6 (0.4; 1.1) |  |
| chronic2 | Absent | 75 (95%) | 29 (97%) | ref | 145 (51%) | 40 (53%) | ref | 438 (26%) | 91 (31%) | ref |  |
| chronic2 | Present | 4 (5%) | 1 (3%) | 0.5 (0; 8.3) | 142 (49%) | 36 (47%) | 1.7 (0.9; 3.3) | 1220 (74%) | 198 (69%) | 0.8 (0.6; 1.0) |  |
| chronic3 | Absent | 78 (99%) | 30 (100%) | ref | 201 (70%) | 57 (75%) | ref | 922 (56%) | 181 (63%) | ref |  |
| chronic3 | Present | 1 (1%) | NA (NA%) | 1.6 (0; Inf) | 86 (30%) | 19 (25%) | 1.2 (0.6; 2.3) | 736 (44%) | 108 (37%) | 0.8 (0.6; 1.0) |  |
| cancer | Absent | - | - | - | 254 (89%) | 71 (93%) | ref | 1413 (85%) | 253 (88%) | ref |  |
| cancer | Present | - | - | - | 33 (11%) | 5 (7%) | 0.6 (0.2; 1.7) | 245 (15%) | 36 (12%) | 0.7 (0.5; 1.1) |  |
| cardiovasc | Absent | 70 (89%) | 29 (97%) | ref | 213 (74%) | 59 (78%) | ref | 603 (36%) | 119 (41%) | ref |  |
| cardiovasc | Present | 9 (11%) | 1 (3%) | 0.4 (0; 4.7) | 74 (26%) | 17 (22%) | 1.0 (0.5; 2.2) | 1054 (64%) | 170 (59%) | 0.8 (0.6; 1.1) |  |
| diabetes | Absent | 78 (99%) | 30 (100%) | ref | 229 (80%) | 63 (83%) | ref | 1067 (64%) | 189 (65%) | ref |  |
| diabetes | Present | 1 (1%) | NA (NA%) | 0 (0; Inf) | 58 (20%) | 13 (17%) | 1.1 (0.5; 2.3) | 591 (36%) | 100 (35%) | 0.9 (0.7; 1.2) |  |
| lungdis | Absent | 67 (85%) | 27 (90%) | ref | 175 (61%) | 49 (65%) | ref | 848 (51%) | 153 (53%) | ref |  |
| lungdis | Present | 12 (15%) | 3 (10%) | 0.5 (0.1; 3.2) | 111 (39%) | 26 (35%) | 1.6 (0.8; 3.0) | 809 (49%) | 136 (47%) | 1.0 (0.8; 1.3) |  |
| obesity | Absent | 75 (95%) | 30 (100%) | ref | 217 (76%) | 61 (80%) | ref | 1306 (79%) | 231 (80%) | ref |  |
| obesity | Present | 4 (5%) | NA (NA%) | 0.6 (0; Inf) | 70 (24%) | 15 (20%) | 1.1 (0.5; 2.2) | 352 (21%) | 58 (20%) | 0.9 (0.7; 1.3) |  |
| rendisease | Absent | 77 (97%) | 27 (90%) | ref | 257 (90%) | 69 (91%) | ref | 1328 (80%) | 243 (84%) | ref |  |
| rendisease | Present | 2 (3%) | 3 (10%) | 60994864.9 (0; Inf) | 30 (10%) | 7 (9%) | 1.3 (0.5; 3.4) | 330 (20%) | 46 (16%) | 0.8 (0.5; 1.1) |  |
| nhosp | 0 | 36 (51%) | 21 (75%) | ref | 171 (59%) | 46 (50%) | ref | 944 (57%) | 219 (67%) | ref |  |
| nhosp | 1 to 2 | 27 (39%) | 5 (18%) | 0.2 (0; 1.1) | 86 (30%) | 32 (35%) | 1.5 (0.8; 2.8) | 548 (33%) | 86 (26%) | 0.6 (0.4; 0.8) |  |
| nhosp | more than 2 | 7 (10%) | 2 (7%) | 0.5 (0; 5.3) | 31 (11%) | 14 (15%) | 2.0 (0.9; 4.4) | 168 (10%) | 21 (6%) | 0.3 (0.2; 0.6) |  |
| gpvisit | 0 | 10 (18%) | 5 (45%) | ref | 32 (22%) | 9 (21%) | ref | 121 (11%) | 34 (19%) | ref |  |
| gpvisit | 1 to 5 | 16 (29%) | 3 (27%) | 0.3 (0; 3.7) | 28 (19%) | 16 (38%) | 2.9 (0.8; 10.2) | 194 (18%) | 29 (16%) | 0.7 (0.3; 1.7) |  |
| gpvisit | more than 5 | 9 (16%) | 3 (27%) | 0.4 (0; 3.6) | 6 (4%) | 2 (5%) | 0.4 (0; 4.2) | 72 (7%) | 16 (9%) | 0.5 (0.1; 1.4) |  |

Chronic: presence of at least one chronic condition; chronic2: presence of at least two chronic conditions; chronic3: presence of at least three chronic conditions; cardiovasc: cardiovascular disease; lungdis: lung disease; rendisease: renal disease; nhosp: number of hospitalizations in previous year; gpvisit: number of general practice visits in previous year.

**Table S5c. Propensity to test-positive among unvaccinated subjects in primary care setting. ORs are adjusted for age, sex, calendar time and season.**

| Primary care |  | Children - unvaccinated | | | Adults - unvaccinated | | | Older adults - unvaccinated | | |
| --- | --- | --- | --- | --- | --- | --- | --- | --- | --- | --- |
| covariate | value | Controls  n(%) | Cases  n(%) | OR  (95%CI) | Controls  n(%) | Cases  n(%) | OR  (95%CI) | Controls  n(%) | Cases  n(%) | OR  (95%CI) |
| chronic | Absent | 1770 (95%) | 2008 (94%) | ref | 1804 (78%) | 1236 (81%) | ref | 71 (34%) | 35 (41%) | ref |
| chronic | Present | 99 (5%) | 120 (6%) | 1.1 (0.8; 1.4) | 509 (22%) | 290 (19%) | 0.8 (0.7; 0.9) | 140 (66%) | 50 (59%) | 0.6 (0.3; 1.2) |
| chronic2 | Absent | 953 (100%) | 1114 (100%) | ref | 1269 (96%) | 791 (97%) | ref | 111 (79%) | 35 (71%) | ref |
| chronic2 | Present | 1 (0%) | NA (NA%) | 0 (0; Inf) | 51 (4%) | 23 (3%) | 0.9 (0.5; 1.6) | 30 (21%) | 14 (29%) | 2.5 (1.0; 6.5) |
| chronic3 | Absent | - | - | - | 1310 (99%) | 810 (100%) | ref | 128 (91%) | 45 (92%) | ref |
| chronic3 | Present | - | - | - | 10 (1%) | 4 (0%) | 0.9 (0.3; 3.0) | 13 (9%) | 4 (8%) | 1.9 (0.5; 8.0) |
| cancer | Absent | 698 (100%) | 720 (100%) | ref | 948 (98%) | 426 (99%) | ref | 90 (87%) | 21 (81%) | ref |
| cancer | Present | 2 (0%) | NA (NA%) | 0 (0; Inf) | 15 (2%) | 6 (1%) | 1.0 (0.4; 2.8) | 14 (13%) | 5 (19%) | 1.8 (0.3; 9.5) |
| cardiovasc | Absent | 679 (100%) | 694 (100%) | ref | 817 (91%) | 394 (93%) | ref | 64 (62%) | 12 (39%) | ref |
| cardiovasc | Present | 2 (0%) | 3 (0%) | 3.3 (0.4; 26.2) | 85 (9%) | 30 (7%) | 0.7 (0.4; 1.2) | 39 (38%) | 19 (61%) | 3.6 (1.1; 11.7) |
| diabetes | Absent | - | - | - | 976 (97%) | 442 (97%) | ref | 91 (81%) | 20 (77%) | ref |
| diabetes | Present | - | - | - | 30 (3%) | 13 (3%) | 1.0 (0.5; 2.2) | 22 (19%) | 6 (23%) | 2.0 (0.4; 9.2) |
| lungdis | Absent | 710 (97%) | 717 (98%) | ref | 923 (91%) | 424 (94%) | ref | 87 (79%) | 22 (85%) | ref |
| lungdis | Present | 19 (3%) | 14 (2%) | 0.6 (0.3; 1.2) | 88 (9%) | 27 (6%) | 0.8 (0.5; 1.4) | 23 (21%) | 4 (15%) | 0.5 (0.1; 2.5) |
| obesity | Absent | 889 (99%) | 1057 (98%) | ref | 1072 (89%) | 688 (89%) | ref | 120 (92%) | 41 (84%) | ref |
| obesity | Present | 11 (1%) | 19 (2%) | 1.3 (0.6; 2.9) | 127 (11%) | 83 (11%) | 0.6 (0.5; 0.9) | 11 (8%) | 8 (16%) | 1.3 (0.4; 4.1) |
| rendisease | Absent | 726 (100%) | 729 (100%) | ref | 999 (100%) | 444 (100%) | ref | 104 (95%) | 21 (95%) | ref |
| rendisease | Present | NA (NA%) | 1 (0%) | 105417.6 (0; Inf) | 4 (0%) | 1 (0%) | 0.7 (0.1; 6.2) | 5 (5%) | 1 (5%) | 1.1 (0; 30.5) |
| nhosp | 0 | 1162 (98%) | 1355 (98%) | ref | 1614 (97%) | 916 (98%) | ref | 146 (91%) | 50 (93%) | ref |
| nhosp | 1 to 2 | 19 (2%) | 25 (2%) | 1.1 (0.6; 2.2) | 40 (2%) | 19 (2%) | 0.8 (0.4; 1.4) | 13 (8%) | 4 (7%) | 0.6 (0.1; 2.5) |
| nhosp | more than 2 | 7 (1%) | NA (NA%) | 0 (0; Inf) | 4 (0%) | 2 (0%) | 0.8 (0.1; 5.1) | 1 (1%) | NA (NA%) | 0 (0; Inf) |
| gpvisit | 0 | 177 (15%) | 201 (15%) | ref | 355 (26%) | 244 (30%) | ref | 11 (10%) | 3 (8%) | ref |
| gpvisit | 1 to 5 | 820 (70%) | 1022 (74%) | 1.2 (1.0; 1.6) | 898 (67%) | 531 (65%) | 0.9 (0.8; 1.2) | 67 (62%) | 24 (67%) | 1.0 (0.2; 5.2) |
| gpvisit | more than 5 | 179 (15%) | 155 (11%) | 1.0 (0.7; 1.5) | 95 (7%) | 45 (5%) | 0.7 (0.5; 1.1) | 30 (28%) | 9 (25%) | 0.9 (0.1; 5.6) |

Chronic: presence of at least one chronic condition; chronic2: presence of at least two chronic conditions; chronic3: presence of at least three chronic conditions; cardiovasc: cardiovascular disease; lungdis: lung disease; rendisease: renal disease; nhosp: number of hospitalizations in previous year; gpvisit: number of general practice visits in previous year.

**Table S5d. Propensity to test-positive among unvaccinated subjects in hospital setting. ORs are adjusted for age, sex, calendar time and season.**

| Hospital |  | Children - unvaccinated | | | Adults - unvaccinated | | | Older adults - unvaccinated | | |
| --- | --- | --- | --- | --- | --- | --- | --- | --- | --- | --- |
| covariate | value | Controls  n(%) | Cases  n(%) | OR  (95%CI) | Controls  n(%) | Cases  n(%) | OR  (95%CI) | Controls  n(%) | Cases  n(%) | OR  (95%CI) |
| chronic | Absent | 1464 (85%) | 951 (84%) | ref | 439 (39%) | 216 (36%) | ref | 122 (10%) | 32 (7%) | ref |
| chronic | Present | 259 (15%) | 184 (16%) | 1.5 (1.2; 1.9) | 693 (61%) | 386 (64%) | 1.2 (0.9; 1.5) | 1117 (90%) | 450 (93%) | 1.4 (0.9; 2.1) |
| chronic2 | Absent | 1640 (99%) | 1055 (99%) | ref | 737 (70%) | 334 (66%) | ref | 432 (37%) | 147 (38%) | ref |
| chronic2 | Present | 21 (1%) | 14 (1%) | 1.8 (0.8; 4.0) | 314 (30%) | 170 (34%) | 1.3 (1.0; 1.7) | 726 (63%) | 244 (62%) | 1.0 (0.8; 1.3) |
| chronic3 | Absent | 1656 (100%) | 1067 (100%) | ref | 888 (84%) | 418 (83%) | ref | 738 (64%) | 270 (69%) | ref |
| chronic3 | Present | 5 (0%) | 2 (0%) | 0.5 (0.1; 3.1) | 163 (16%) | 86 (17%) | 1.2 (0.9; 1.7) | 420 (36%) | 121 (31%) | 0.7 (0.6; 1.0) |
| cancer | Absent | 1652 (99%) | 1065 (100%) | ref | 968 (92%) | 472 (94%) | ref | 960 (83%) | 342 (87%) | ref |
| cancer | Present | 9 (1%) | 4 (0%) | 0.4 (0.1; 1.5) | 83 (8%) | 32 (6%) | 0.8 (0.5; 1.2) | 197 (17%) | 49 (13%) | 0.6 (0.4; 0.9) |
| cardiovasc | Absent | 1629 (98%) | 1046 (98%) | ref | 872 (83%) | 390 (77%) | ref | 499 (43%) | 157 (40%) | ref |
| cardiovasc | Present | 32 (2%) | 23 (2%) | 1.6 (0.9; 3.0) | 179 (17%) | 114 (23%) | 1.6 (1.2; 2.2) | 658 (57%) | 234 (60%) | 1.1 (0.8; 1.4) |
| diabetes | Absent | 1656 (100%) | 1060 (99%) | ref | 946 (90%) | 440 (87%) | ref | 795 (69%) | 297 (76%) | ref |
| diabetes | Present | 5 (0%) | 9 (1%) | 4.1 (1.3; 13.4) | 105 (10%) | 64 (13%) | 1.3 (0.9; 1.9) | 363 (31%) | 94 (24%) | 0.7 (0.5; 0.9) |
| lungdis | Absent | 1604 (97%) | 1036 (97%) | ref | 832 (79%) | 395 (78%) | ref | 754 (65%) | 245 (63%) | ref |
| lungdis | Present | 57 (3%) | 33 (3%) | 1.2 (0.7; 2.0) | 219 (21%) | 109 (22%) | 1.3 (0.9; 1.7) | 404 (35%) | 146 (37%) | 1.2 (0.9; 1.5) |
| obesity | Absent | 1568 (94%) | 1056 (99%) | ref | 880 (84%) | 420 (83%) | ref | 946 (82%) | 325 (83%) | ref |
| obesity | Present | 92 (6%) | 13 (1%) | 0.5 (0.3; 1.1) | 170 (16%) | 84 (17%) | 1.3 (0.9; 1.7) | 212 (18%) | 66 (17%) | 1.1 (0.8; 1.6) |
| rendisease | Absent | 1647 (99%) | 1053 (99%) | ref | 994 (95%) | 468 (93%) | ref | 946 (82%) | 338 (86%) | ref |
| rendisease | Present | 14 (1%) | 16 (1%) | 2.1 (0.9; 4.8) | 57 (5%) | 36 (7%) | 1.4 (0.9; 2.3) | 212 (18%) | 53 (14%) | 0.6 (0.5; 0.9) |
| nhosp | 0 | 467 (60%) | 416 (61%) | ref | 573 (64%) | 310 (65%) | ref | 599 (57%) | 222 (56%) | ref |
| nhosp | 1 to 2 | 249 (32%) | 203 (30%) | 0.8 (0.6; 1.0) | 236 (26%) | 132 (28%) | 1.0 (0.7; 1.3) | 360 (34%) | 141 (36%) | 0.9 (0.7; 1.2) |
| nhosp | more than 2 | 57 (7%) | 59 (9%) | 0.9 (0.6; 1.3) | 82 (9%) | 36 (8%) | 0.8 (0.5; 1.3) | 90 (9%) | 33 (8%) | 1.0 (0.6; 1.5) |
| gpvisit | 0 | 119 (15%) | 120 (22%) | ref | 223 (39%) | 84 (31%) | ref | 138 (20%) | 57 (24%) | ref |
| gpvisit | 1 to 5 | 446 (55%) | 331 (62%) | 0.6 (0.4; 0.8) | 158 (27%) | 109 (40%) | 1.8 (1.2; 2.6) | 188 (27%) | 75 (32%) | 0.8 (0.5; 1.3) |
| gpvisit | more than 5 | 113 (14%) | 68 (13%) | 0.4 (0.3; 0.7) | 22 (4%) | 25 (9%) | 1.6 (0.8; 3.4) | 40 (6%) | 32 (13%) | 1.1 (0.6; 2.3) |

Chronic: presence of at least one chronic condition; chronic2: presence of at least two chronic conditions; chronic3: presence of at least three chronic conditions; cardiovasc: cardiovascular disease; lungdis: lung disease; rendisease: renal disease; nhosp: number of hospitalizations in previous year; gpvisit: number of general practice visits in previous year.

SUPPLEMENT 6: REFERENCE MODEL VS. UNADJUSTED MODEL

Table S6a. Absolute and relative changes in IVE estimate when adjusting for age, sex, calendar time and season (i.e. the reference model) compared to the model adjust for season only, by age group in the primary care setting.

|  | Children | | Adults | | Older adults | |
| --- | --- | --- | --- | --- | --- | --- |
|  | Absolute % Δ | Relative % Δ (ref=1.0) | Absolute % Δ | Relative % Δ (ref=1.0) | Absolute % Δ | Relative % Δ (ref=1.0) |
| Age | 1.0 (-3.8; 5.7) | 1.03 (0.92; 1.16) | -0.5 (-8.8; 7.8) | 1.01 (0.89; 1.14) | -2.0 (-9.2; 5.2) | 0.98 (0.95; 1.00) |
| Sex | 0.1 (-0.6; 0.8) | 1.00 (0.98; 1.02) | -0.3 (-1.4; 0.9) | 1.00 (0.97; 1.02) | 1.8 (-31.9; 35.5) | 1.01 (0.94; 1.10) |
| Time | -0.2 (-30.5; 30.1) | **1.16 (0.89; 1.52)** | 4.0 (-6.5; 14.5) | 1.08 (0.92; 1.28) | 22.4 (21.7; 23.1) | **1.24 (1.13; 1.37)** |
| Age, sex, calendar time | 0.5 (-32.5; 33.5) | **1.20 (0.86; 1.66)** | 2.2 (-17.3; 21.8) | 1.07 (0.79; 1.43) | 23.7 (-50.8; 98.3) | **1.26 (1.11; 1.43)** |

Table S6b. Absolute and relative changes in IVE estimate when adjusting for age, sex, calendar time and season (i.e. the reference model) compared to the model adjust for season only, by age group in the hospital setting.

|  | Children | | Adults | | Older adults | |
| --- | --- | --- | --- | --- | --- | --- |
|  | Absolute % Δ | Relative % Δ (ref=1.0) | Absolute % Δ | Relative % Δ (ref=1.0) | Absolute % Δ | Relative % Δ (ref=1.0) |
| Age | 6.5 (-14.1; 27.1) | 1.08 (0.84; 1.39) | 0.9 (-1.4; 3.1) | 1.01 (0.98; 1.05) | 0.9 (-1.4; 3.1) | 1.00 (0.53; 1.90) |
| Sex | -0.1 (-0.8; 0.6) | 1.00 (0.99; 1.01) | 0.6 (-0.7; 1.8) | 1.01 (1.00; 1.02) | 7.8 (-11.7; 27.3) | NA (NA; NA) |
| Calendar time | -10.7 (-37.1; 15.8) | 0.87 (0.57; 1.32) | -3.6 (-10.9; 3.7) | 0.96 (0.90; 1.04) | 5.5 (0.1; 10.9) | NA (NA; NA) |
| Age, sex, calendar time | -5.4 (-40.4; 29.6) | NA (NA; NA) | -3.2 (-10.2; 3.8) | 0.96 (0.87; 1.06) | 4.1 (-0.4; 8.6) | NA (NA; NA) |

NA: not available

SUPPLEMENT 7: SENSITIVITY ANALYSIS

**Table S7. Sensitivity analysis: relative changes in IVE estimate when adjusting for an additional covariate compared to the reference model adjusted for age, sex, calendar, season *and chronic conditions*; by age group and setting.**

|  | Relative change in IVE | | | | | |
| --- | --- | --- | --- | --- | --- | --- |
|  | Primary care | | | Hospital | | |
| Covariate | Children | Adults | Older adults | Children | Adults | Older adults |
| chronic | 1.00 (0.98; 1.02) | 0.97 (0.86; 1.10) | 0.97 (0.86; 1.11) | 1.08 (0.63; 1.87) | 1.01 (0.94; 1.08) | 1.01 (0.99; 1.03) |
| chronic2 | NA (NA; NA) | 1.00 (0.97; 1.03) | 1.01 (0.94; 1.09) | 1.01 (0.96; 1.07) | 0.99 (0.96; 1.02) | 1.01 (0.98; 1.03) |
| chronic3 | NA (NA; NA) | 1.00 (0.98; 1.02) | 0.99 (0.92; 1.07) | 0.99 (0.93; 1.05) | 1.01 (0.98; 1.04) | 1.00 (0.99; 1.01) |
| cancer | 0.99 (0.97; 1.01) | 1.00 (0.99; 1.01) | 1.01 (0.94; 1.09) | 0.98 (0.74; 1.29) | 0.98 (0.98; 0.99) | 0.99 (0.98; 1.00) |
| cardiovasc | 0.96 (0.91; 1.01) | 1.01 (0.92; 1.10) | 0.95 (0.86; 1.09) | 1.02 (1.00; 1.05) | 1.00 (0.98; 1.02) | 1.01 (0.99; 1.03) |
| diabetes | NA (NA; NA) | 1.01 (0.84; 1.21) | 1.00 (0.93; 1.09) | 1.01 (1.00; 1.01) | 1.00 (0.97; 1.03) | 1.00 (0.98; 1.02) |
| lungdis | 0.99 (0.96; 1.03) | 0.97 (0.80; 1.18) | 1.12 (0.92; 1.30) | 1.00 (0.67; 1.49) | 1.00 (0.98; 1.03) | 1.00 (0.99; 1.01) |
| obesity | 1.00 (1.00; 1.00) | 1.00 (0.97; 1.03) | 1.02 (0.99; 1.05) | 0.99 (0.96; 1.02) | 0.99 (0.96; 1.01) | 1.00 (0.98; 1.02) |
| rendisease | NA (NA; NA) | 1.00 (0.93; 1.07) | 1.00 (0.94; 1.04) | 0.98 (0.88; 1.10) | 1.00 (0.99; 1.01) | 0.99 (0.96; 1.02) |
| nhosp | 1.00 (0.93; 1.08) | 0.99 (0.86; 1.15) | 1.01 (0.95; 1.07) | 0.95 (0.89; 1.02) | 0.98 (0.95; 1.02) | 1.00 (0.96; 1.04) |
| gpvisit | 0.98 (0.94; 1.03) | 1.03 (0.99; 1.07) | 0.99 (0.90; 1.08) | 1.01 (0.79; 1.27) | 0.97 (0.82; 1.15) | 1.03 (0.95; 1.11) |

Chronic: presence of at least one chronic condition; chronic2: presence of at least two chronic conditions; chronic3: presence of at least three chronic conditions; cardiovasc: cardiovascular disease; lungdis: lung disease; rendisease: renal disease; nhosp: number of hospitalizations in previous year; gpvisit: number of general practice visits in previous year.

SUPPLEMENT 8: PROPENSITY-SCORE MODEL METHODS

The propensity score method was based on inverse probability weighting. Following Vanderweele et al. (https://doi.org/10.1093/aje/kwr334), we assumed that the population propensity score could be well approximated by the vaccination propensity among the test-negative controls (and hence making a ‘rare disease’ assumption). The covariates included in the propensity score models were modelled using the same GAM approach as for the conditional logistic regression models with vaccination status as outcome. Weights larger than the 95% percentile were set equal to the 95% percentile. Note that similarly to the conditional logistic regression approach also here a two stage method was used, i.e., the marginal effects were estimated for each site and subsequently pooled.

SUPPLEMENT 9: E-VALUES

Table S9. E-values for pooled VE point estimate and corresponding lower bound of 95%CI

| Age group | Setting | VE | E-value for VE | Lower 95%CI limit of VE | E-value for lower limit |
| --- | --- | --- | --- | --- | --- |
| Children | Primary Care | 56.5 | 4.0 | -14.1 | 1.0 |
| Adults | Primary Care | 36.4 | 2.5 | 14.3 | 1.6 |
| Older adults | Primary Care | 6.3 | 1.3 | -22.2 | 1.0 |
| Children | Hospital | 24.8 | 2.0 | -2.3 | 1.0 |
| Adults | Hospital | 34.3 | 2.4 | -2.2 | 1.0 |
| Older adults | Hospital | 38.3 | 2.6 | 26.6 | 2.1 |

CI: confidence interval; VE: vaccine effectiveness
